# SYK inhibition targets acute myeloid leukemia stem cells by blocking their oxidative metabolism

**DOI:** 10.1038/s41419-020-03156-8

**Published:** 2020-11-06

**Authors:** Anna Polak, Emilia Bialopiotrowicz, Beata Krzymieniewska, Jolanta Wozniak, Marta Stojak, Magdalena Cybulska, Ewelina Kaniuga, Michał Mikula, Ewa Jablonska, Patryk Gorniak, Monika Noyszewska-Kania, Maciej Szydlowski, Karolina Piechna, Katarzyna Piwocka, Lukasz Bugajski, Ewa Lech-Maranda, Joanna Barankiewicz, Agnieszka Kolkowska-Lesniak, Elzbieta Patkowska, Eliza Glodkowska-Mrowka, Natalia Baran, Przemyslaw Juszczynski

**Affiliations:** 1 Department of Experimental Hematology, Institute of Hematology and Transfusion Medicine, Warsaw, Poland; 2 Department of Diagnostic Hematology, Institute of Hematology and Transfusion Medicine, Warsaw, Poland; 3 Jagiellonian Centre for Experimental Therapeutics (JCET), Jagiellonian University, Kraków, Poland; 4 Department of Genetics, Maria Sklodowska-Curie National Research Institute of Oncology, Warsaw, Poland; 5 Laboratory of Cytometry, Nencki Institute of Experimental Biology, Polish Academy of Sciences, Warsaw, Poland; 6 Department of Hematology, Institute of Hematology and Transfusion Medicine, Warsaw, Poland; 7 Department of Leukemia, The University of Texas MD Anderson Cancer Center, Houston, TX USA

**Keywords:** Cancer stem cells, Translational research, Oncogenesis

## Abstract

Spleen tyrosine kinase (SYK) is an important oncogene and signaling mediator activated by cell surface receptors crucial for acute myeloid leukemia (AML) maintenance and progression. Genetic or pharmacologic inhibition of SYK in AML cells leads to increased differentiation, reduced proliferation, and cellular apoptosis. Herein, we addressed the consequences of SYK inhibition to leukemia stem-cell (LSC) function and assessed SYK-associated pathways in AML cell biology. Using gain-of-function MEK kinase mutant and constitutively active STAT5A, we demonstrate that R406, the active metabolite of a small-molecule SYK inhibitor fostamatinib, induces differentiation and blocks clonogenic potential of AML cells through the MEK/ERK1/2 pathway and STAT5A transcription factor, respectively. Pharmacological inhibition of SYK with R406 reduced LSC compartment defined as CD34^+^CD38^−^CD123^+^ and CD34^+^CD38^−^CD25^+^ in vitro, and decreased viability of LSCs identified by a low abundance of reactive oxygen species. Primary leukemic blasts treated ex vivo with R406 exhibited lower engraftment potential when xenotransplanted to immunodeficient NSG/J mice. Mechanistically, these effects are mediated by disturbed mitochondrial biogenesis and suppression of oxidative metabolism (OXPHOS) in LSCs. These mechanisms appear to be partially dependent on inhibition of STAT5 and its target gene MYC, a well-defined inducer of mitochondrial biogenesis. In addition, inhibition of SYK increases the sensitivity of LSCs to cytarabine (AraC), a standard of AML induction therapy. Taken together, our findings indicate that SYK fosters OXPHOS and participates in metabolic reprogramming of AML LSCs in a mechanism that at least partially involves STAT5, and that SYK inhibition targets LSCs in AML. Since active SYK is expressed in a majority of AML patients and confers inferior prognosis, the combination of SYK inhibitors with standard chemotherapeutics such as AraC constitutes a new therapeutic modality that should be evaluated in future clinical trials.

## Introduction

Acute myeloid leukemia (AML) is a heterogeneous and aggressive malignancy characterized by uncontrolled proliferation, increased survival, and impaired differentiation of hematopoietic stem and progenitor cells^1^. Despite recent approvals of new targeted agents, intensive “3 + 7” chemotherapy with anthracycline and cytarabine (AraC) remains the standard of care for a majority of AML patients. Despite initial responses to this regimen, most patients eventually relapse and long-term prognosis for AML patients is very poor^2–6^. The therapy-resistant, persistent cells that give rise to relapses are believed to reside in rare leukemia stem-cell (LSCs) population; accordingly, the LSCs frequency dramatically increases after treatment^7^. Therefore, targeting LSCs is a promising therapeutic strategy in AML, potentially eliminating the roots of the disease.

LSCs are typically characterized by their quiescent nature, immature phenotype, distinct metabolic features, and reliance on certain signaling pathways. For example, several lines of evidence point toward an increased LSCs dependence on oxidative metabolism and efficient management of oxidative stress. Indeed, inhibition of oxidative metabolism and/or induction of oxidative stress in LSCs leads selectively to LSCs death^8–10^, indicating that these features can be exploited therapeutically. However, cell-intrinsic signaling circuits that induce/maintain LSCs metabolic characteristics are less well defined. Identification of signaling pathways responsible for metabolic reprogramming in LSCs might provide additional therapeutic targets, perhaps with broader therapeutic windows than direct inhibition of mitochondrial oxidative phosphorylation (OXPHOS).

Spleen tyrosine kinase (SYK) activity and oncogenic properties in AML were first discovered in a study integrating chemical, proteomic, and genomic approaches to identify new therapeutic strategies^11,12^. In AML patients, SYK abundance and activity are associated with an unfavorable outcome independent of age, cytogenetics, and leukocyte count^13^. SYK phosphorylation and activity is induced by multiple upstream signaling pathways critical for AML development, including integrin signaling and FLT3-ITD, indicating that SYK is a central signaling hub and a mediator of multiple pathways that drive AML^13–17^.

Once activated, SYK regulates many downstream signaling pathways crucial for cell survival, including mTOR and MEK/ERK1/2, cooperates with FLT3-ITD to activate MYC transcriptional program, and directly interacts with and activates STAT5 and STAT3 transcription factors^14–16,18,19^. Consistently, SYK inhibition with R406, an active metabolite of FDA-approved, ATP-competitive SYK inhibitor fostamatinib, with less activity reported for FLT3, c-Kit, and Lck, induces differentiation and apoptosis of leukemic cells^11,20,21^.

Given the activation of SYK in AML and profound consequences of its inhibition, we dissected the role of SYK-downstream signaling pathways in cell differentiation and LSCs function. We characterize the role of SYK-dependent MEK/ERK1/2 signaling in differentiation and demonstrate that SYK blockade decreases mitochondrial biogenesis and function in a mechanism involving attenuation of STAT5 and MYC activity, leading to decreased oxidative metabolism and LSCs death.

## Results

### SYK and SYK-initiated downstream signaling in AML

**Fig. 1:**
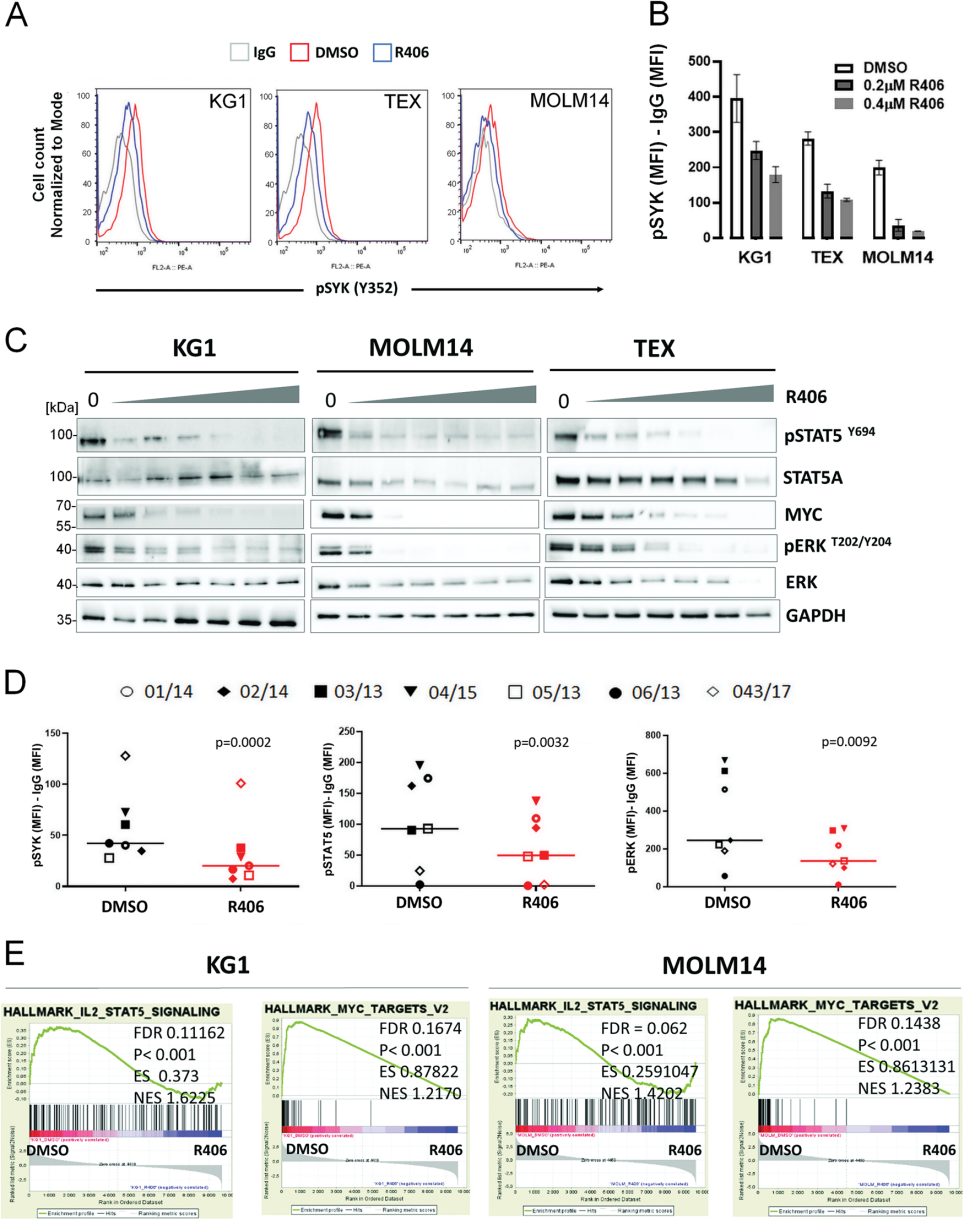
R406 inhibits SYK and SYK-dependent signaling pathways in acute myeloid leukemia (AML) cells. **A** Representative histograms showing SYK phosphorylation status (Tyr352) in AML cell lines (KG1, MOLM14 and TEX) at baseline and after incubation with R406 (0.2 µM, 24 h). SYK phosphorylation was assessed by intracellular phospho-specific flow cytometry. **B** Quantification of changes in SYK activity from panel **A**. SYK Tyr352 mean fluorescence intensities (MFI) after subtracting MFI of isotype control are shown. Bars represent mean + /− SD from two biological replicates. **C** KG1, MOLM14, and TEX cells were incubated for 24 h with increasing doses of R406 (0.075 µM, 0.2 µM, 0.4 µM, 0.6 µM, 1 µM, and 4 µM). Thereafter, the phosphorylation status of ERK1/2, STAT5, and the level of MYC protein were assessed by immunoblotting. **D** Primary AML blasts from seven patients were treated with R406 (1 µM) and assessed by phosflow. pSYK, p-STAT5, and pERK MFI values of individual primary samples, after subtracting the MFI of IgG control, before and after R406 treatment are shown. *P* values were calculated using paired *t* test. **E** GSEA plots showing downregulation of IL2-STAT5 components and MYC targets in KG1 and MOLM14 cell lines after R406 treatment. Data were derived from the publicly accessible dataset available from GEO at the accession number GSE46302. FDR: false discovery rates, ES: enrichment score, NES: normalized enrichment score.

To better understand SYK-regulated cellular signaling in AML, we first assessed the phosphorylation/activation status of SYK and SYK-downstream pathways at baseline and after incubation with R406. Tyrosine 352, associated with SYK activation, was phosphorylated in the majority of tested cell lines and primary patient-derived blasts (Supplementary Fig. S1A). SYK phosphorylation was markedly reduced by R406 in all primary AML samples tested (*n* = 7) and in TEX, KG1, and MOLM14 cell lines (Fig. 1A, B, D). Given the highest SYK activity and sensitivity to R406 in TEX, KG1, and MOLM14, as compared to other cells (Supplementary Fig. S1B), those lines were selected for subsequent experiments. Next, we evaluated the impact of R406 on the activity of SYK-downstream signaling pathways ERK1/2 and STAT5, which are a part of SYK interactome in AML^14,16^. In addition, we also assessed the expression levels of MYC, a STAT5 target gene^22^. Incubation with R406 dose-dependently inhibited p-ERK1/2^T202/Y204^, p-STAT5^Y694^, and decreased MYC levels (Fig. 1C) in AML cells lines, and decreased p-ERK1/2^T202/Y204^ and p-STAT5^Y694^ in primary AML blasts (Fig. 1D). Of note, P505 and entospletinib, inhibitors highly specific for SYK, phenocopied the results obtained with R406 in KG1, MOLM14, and TEX cells (Supplementary Fig. S1F, G). In line with these findings, gene set enrichment analysis (GSEA) of publicly available microarray data^15^ revealed that multiple components of IL2-STAT5 signaling and MYC target genes were among the most downregulated gene sets in AML cell lines after R406 treatment (Fig. 1E). Parallel with the reduction of p-ERK1/2, p-STAT5, and MYC levels, SYK inhibition profoundly decreased clonogenic potential and induced differentiation in AML cell lines (Supplementary Fig. S1C–E). Taken together, these results indicate that in AML, SYK inhibition reduces the activity of its downstream pathways (p-ERK1/2, p-STAT5, and MYC), induces differentiation, and reduces clonogenic potential and viability of AML cells.

### SYK signals through ERK1/2 to block differentiation of AML cells

**Fig. 2:**
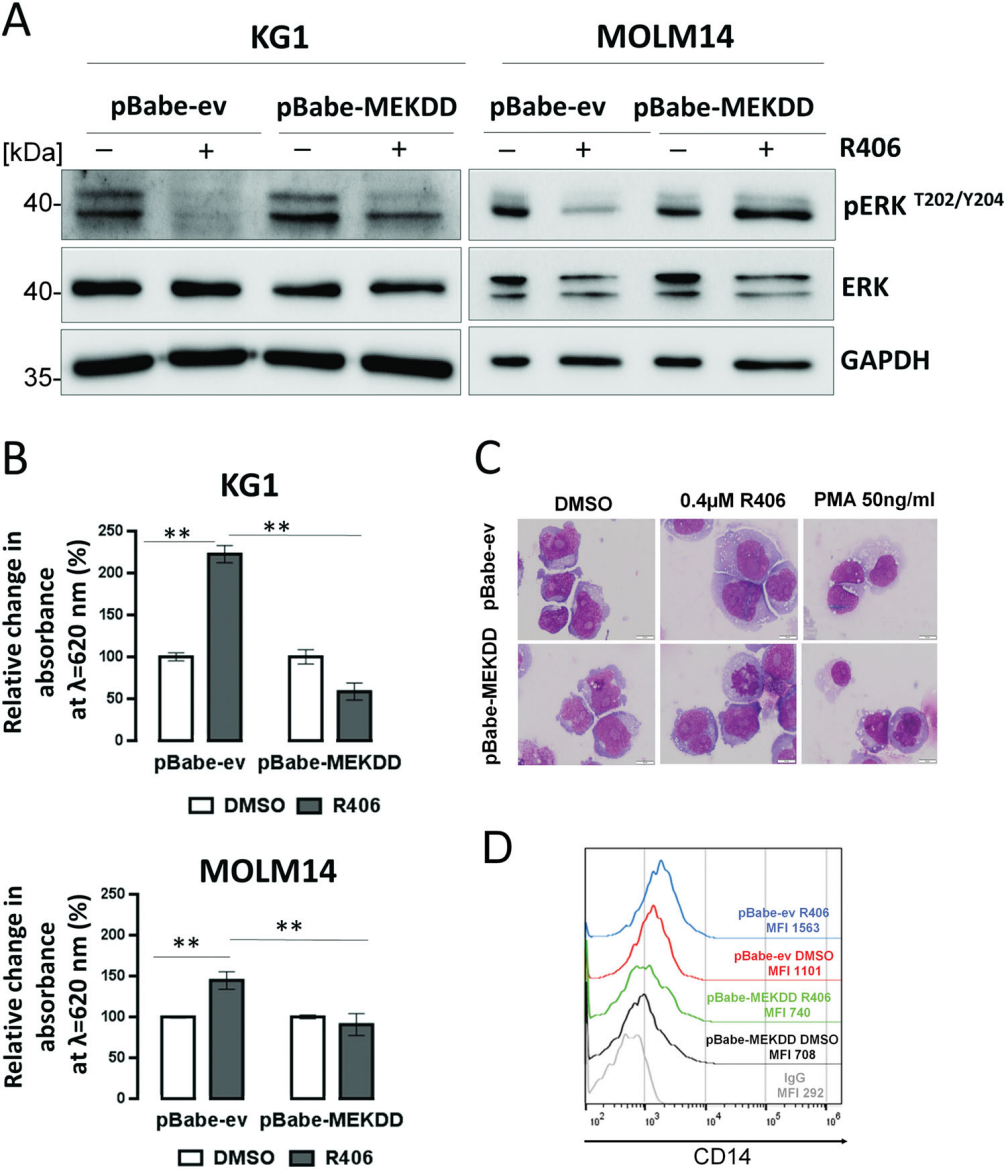
SYK signals through the MAPK/ERK1/2 pathway to block differentiation of leukemic cells. **A** KG1 and MOLM14 cells transduced with an empty vector or a vector containing constitutively active form of MEK1 kinase (MEK-DD) were treated with R406 (KG1: 4 µM, MOLM14: 0.075 µM) for 24 h; thereafter, the phosphorylation status of ERK1/2 was assessed by immunoblotting. **B** Transfected cells were incubated for 3 days with R406 (KG1 0.4 µM, MOLM14 0.075 µM), and NBT reduction was assessed. The graph shows a relative change in absorbance at 620 nm. The experiment was repeated twice. Bars indicate mean + /− SD from biological replicates (*n* = 2) of a representative experiment. **P < 0.01. **C** May–Grunwald–Giemsa staining of KG1 cells containing either MEK-DD or empty vector after 5 days of treatment with R406 or PMA at the indicated concentration. Differentiated cells are morphologically characterized by nuclear condensation, increased cytoplasm/nucleus ratio, increased cell volume, and increased number of cytoplasmic granules. **D** KG1 cells containing either MEK-DD or empty vector were treated with R406 (0.4 µM) for 3 days. Thereafter, CD14 level was assessed by flow cytometry.

Since activated ERK1/2 phosphorylates CCAAT/enhancer-binding protein α (C/EBPα) on serine 21 and inhibits activity of this myeloid differentiation transcription factor^23^, we hypothesized that the aberrant activation of the MEK/ERK1/2 pathway through SYK might contribute to the differentiation blockade in AML cells. To test this hypothesis, we retrovirally transduced KG1 and MOLM14 cell lines with a constitutively active form of an upstream MEK1 kinase (MEK-DD)^24,25^ and assessed the differentiation status of cells incubated either with R406 or DMSO. Consistent with previous results, in cells expressing empty vector, R406 treatment markedly reduced the p-ERK1/2 level, enhanced superoxide production, increased CD14 surface level and expression of genes involved in myeloid maturation, and increased the number of cells with morphological signs of differentiation (Fig. 2 and Supplementary Fig. S2). In contrast, in MEK-DD-transduced cells, R406 only moderately reduced the level of p-ERK1/2, and MEK-DD cells treated with R406 did not exhibit features of differentiation (Fig. 2A–D and Supplementary Fig. S2). These data indicate that MEK/ERK1/2 pathway activation downstream of SYK plays an important role in differentiation arrest in AML cells. Reduced MEK/ERK1/2 activity after R406 treatment is responsible for the induction of myeloid maturation.

### SYK-dependent p-STAT5 activity is required to maintain the clonogenic potential of AML cells

**Fig. 3:**
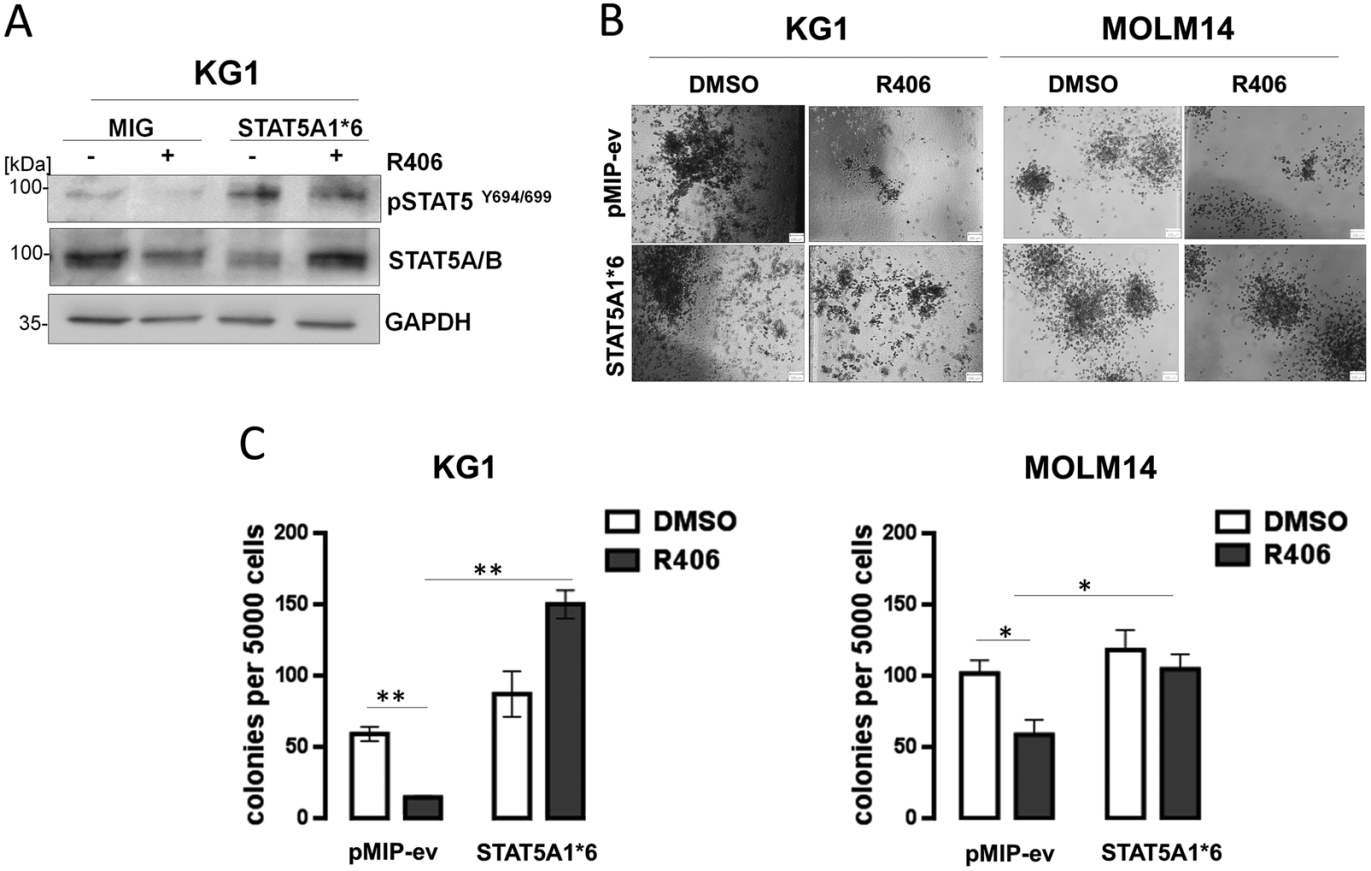
SYK-dependent p-STAT5 activity controls the clonogenic potential of acute myeloid leukemia (AML) cells. **A** KG1 cells were electroporated with either an empty vector (pMIG) or a vector containing constitutively active form of STAT5 (STAT5A1*6). Forty-eight hours later, cells were incubated with 0.4 µM R406 for additional 48 h and assessed by western blotting to determine the phosphorylation status of STAT5. **B**, **C** KG1 or MOLM14 cells transduced with either STAT5A1*6 or an empty vector were incubated with R406 (0.4 μM) and assessed for the ability to form colonies in methylcellulose. The graph shows the mean number of colonies + /− SD, counted 14 days after plating (averaged data from three independent plates). *P* value was calculated using Student’s *t* test. *P < 0.05, **P < 0.01.

STAT5 activity is important for the regulation of self-renewal of hematopoietic stem cells and LSCs^26–29^. Since R406 reduces STAT5 activity, we hypothesized that in AML cells with active SYK, STAT5 might be SYK’s downstream mediator of the stem-cell features. To test this hypothesis, we retrovirally transduced KG1 and MOLM14 cell lines with an empty vector or a vector containing constitutively active form of STAT5A (STAT5A1*6) that mediates a high level of transcription activation independent of cytokine stimulation^30,31^. Thereafter, transduced KG1 and MOLM14 cells were incubated with R406 and colony formation in semi-solid methylcellulose was assessed. As expected, R406 did not block STAT5A1*6 activity, indicated by unchanged p-STAT5^Y694/699^ levels (Fig. 3A). R406 treatment decreased colony formation capacity of the empty vector containing cells but had no significant effect on the colony-forming ability of STAT5A1*6-expressing cells (Fig. 3B, C). These experiments demonstrate that R406-driven reduction of clonogenic potential in AML cells is mediated by decreased STAT5 activity.

### SYK inhibition targets LSC-enriched AML subpopulations

**Fig. 4:**
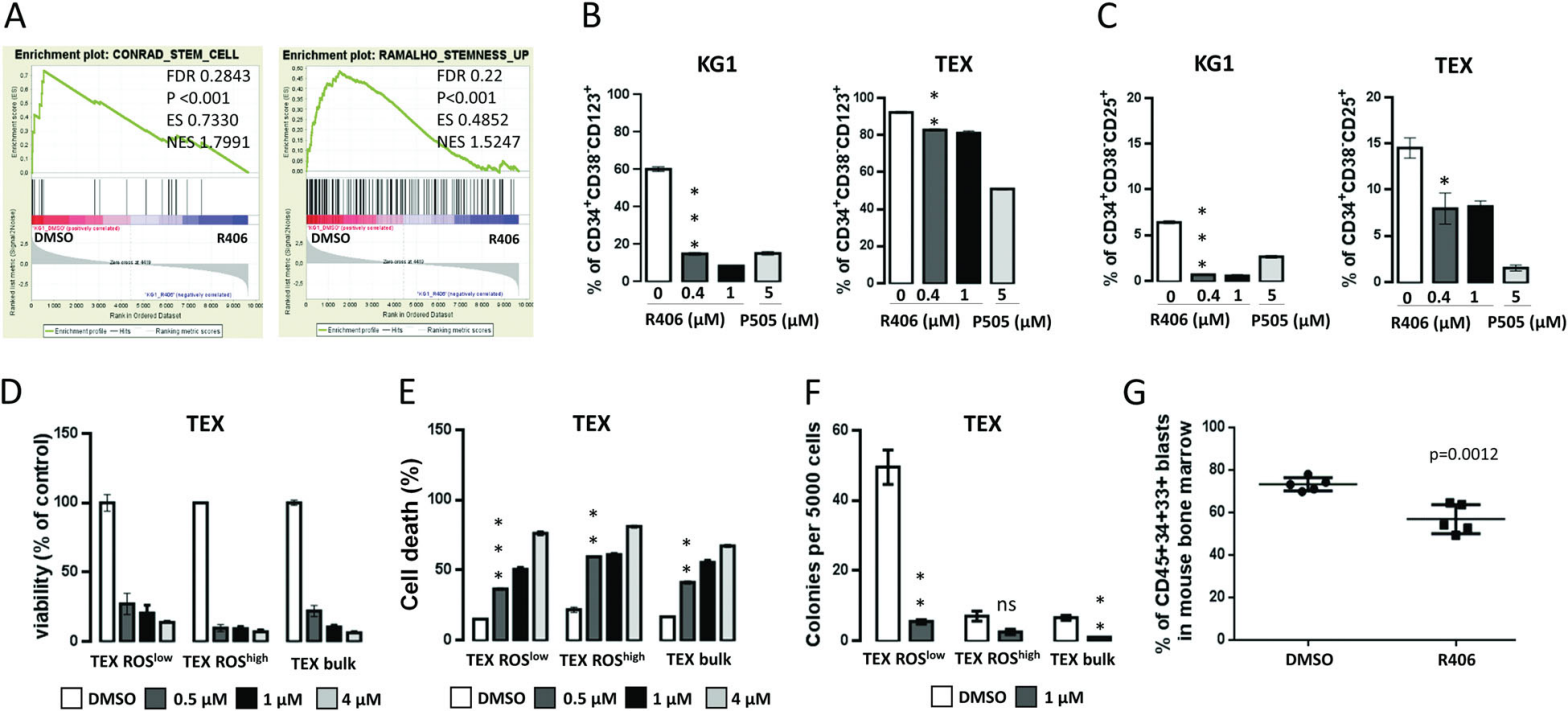
SYK inhibition targets leukemia stem cells (LSC)-enriched acute myeloid leukemia (AML) subpopulations. **A** GSEA plots showing downregulation of stem-cell transcriptional program in KG1 and MOLM14 cell lines after R406 treatment. Source data were derived from the publicly accessible dataset available from GEO at the accession number GSE46302. **B**, **C** KG1 and TEX cells were incubated with the indicated concentration of R406 or P505 for 48 h, then the expression level of either CD25 or CD123 in CD34^+^CD38^−^-gated population was assessed by flow cytometry. The experiment was repeated twice. Bars represent mean + /− SD from two biological replicates of a representative experiment. *P* value was calculated using Student’s *t* test. *P < 0.05, **P < 0.01, ***P < 0.001. **D**, **E** Sorted ROS-low, ROS-high and bulk TEX cells were treated with vehicle or increasing concentration of R406 for 3 days. Thereafter, cells were analyzed by MTS assay to assess cell proliferation (**D**), or stained with PI followed by flow-cytometry analysis to assess cell death (**E**). In **D**, the results are shown relative to DMSO-treated control and represent mean + /− SD from three biological replicates. In **E**, the percentage of PI-positive cells is shown. The experiment was repeated twice. Bars represent mean + /− SD from two biological replicates of a representative experiment. **P < 0.01, ***P < 0.001. **F** In total, 5 × 10^3^ of sorted ROS-low, ROS-high, and bulk TEX cells were plated in GFH4434 medium containing either DMSO or R406 (0.17 µM). Colonies consisting of a minimum of ten cells were counted 14 days after plating. Graph shows the mean number of colonies (+/− SD) obtained from two independent plates. **P < 0.01. **G** Primary AML blasts (from patient 096/17) were cultured ex vivo for 24 h with DMSO or 4 µM of R406. Thereafter leukemia cells were transplanted into NSG/J mice (*n* = 5 per group). Mice were killed 8 weeks after transplantation, and AML engraftment was assessed by FACS analysis for the presence of human CD45^+^CD33^+^CD34^+^ cells in mouse bone marrow. Statistical analysis was performed using Student’s *t* test.

SYK is a critical mediator of integrin signaling in AML. Since the loss of integrin β3 (ITGB3) downregulates LSC transcriptional program^15^, we hypothesized that the loss of SYK activity might also eliminate LSCs in AML. Accordingly, GSEA of publicly available gene expression datasets revealed marked and coherent downregulation of stemness-related genes in KG1 cells after R406 treatment (Fig. 4A). In KG1 and TEX cells treated with R406, an abundance of LSC-enriched CD34^+^CD38^−^CD25^+^ and CD34^+^CD38^-^CD123^+^ leukemic populations markedly decreased, further supporting the role of SYK in LSC maintenance^32,33^ (Fig. 4B, C).

Since functionally defined LSCs in AML are characterized by a low rate of energy metabolism and low levels of reactive oxygen species, we further tested the R406 effects on sorted ROS-low AML cells^10^. For these experiments, we used TEX line, given its hierarchical organization similar to normal hematopoiesis and AML^34^. First, TEX cells were sorted to obtain subsets with low and high endogenous ROS levels (ROS-low and ROS-high cells). The stem-cell features of TEX ROS-low fraction were confirmed by their higher colony-forming capacity and their quiescent nature (higher fraction of G0 cells and lower Ki-67 staining, compared to corresponding ROS-high cells, Supplementary Fig. S3A–C). To test whether R406 affects the viability of the LSC-enriched ROS-low population, we exposed this leukemic fraction to increasing doses of R406. SYK inhibition reduced proliferation, clonogenic potential in the serial replating assay, and increased cell death in a dose-dependent manner. Of note, R406 exhibited even higher toxicity in ROS-high populations, indicating that SYK inhibition targets LSCs and their progeny (Fig. 4D–F and Supplementary Fig. S3D). P505 exhibited similar effects and similarly decreased TEX ROS-low viability (Supplementary Fig. S4).

To further confirm that SYK inhibition targets LSCs, we measured the ability of primary AML blasts cultured ex vivo with R406, to engraft in immunodeficient NSG/J mice. For this purpose, primary AML cells were cultured with R406 or DMSO for 24 h, and 10^5^ of viable cells were transplanted into NSG/J mice via tail vein injection. Eight weeks later, the mice transplanted with AML cells cultured ex vivo with R406 exhibited significantly lower fraction of leukemia cells (hCD45^+^hCD34^+^hCD33^+^) within the bone marrow, when compared to mice injected with control primary AML blasts (Fig. 4G). In a secondary transplant, mice receiving bone marrow cells from donors that had been inoculated with R406-pretreated AMLs cells showed a trend to a reduced leukemia burden (Supplementary Fig. S3E). These results further demonstrate that SYK contributes to the maintenance of LSCs functionality in AML.

### SYK inhibition decreases mitochondrial biogenesis and OXPHOS metabolism in AML

**Fig. 5:**
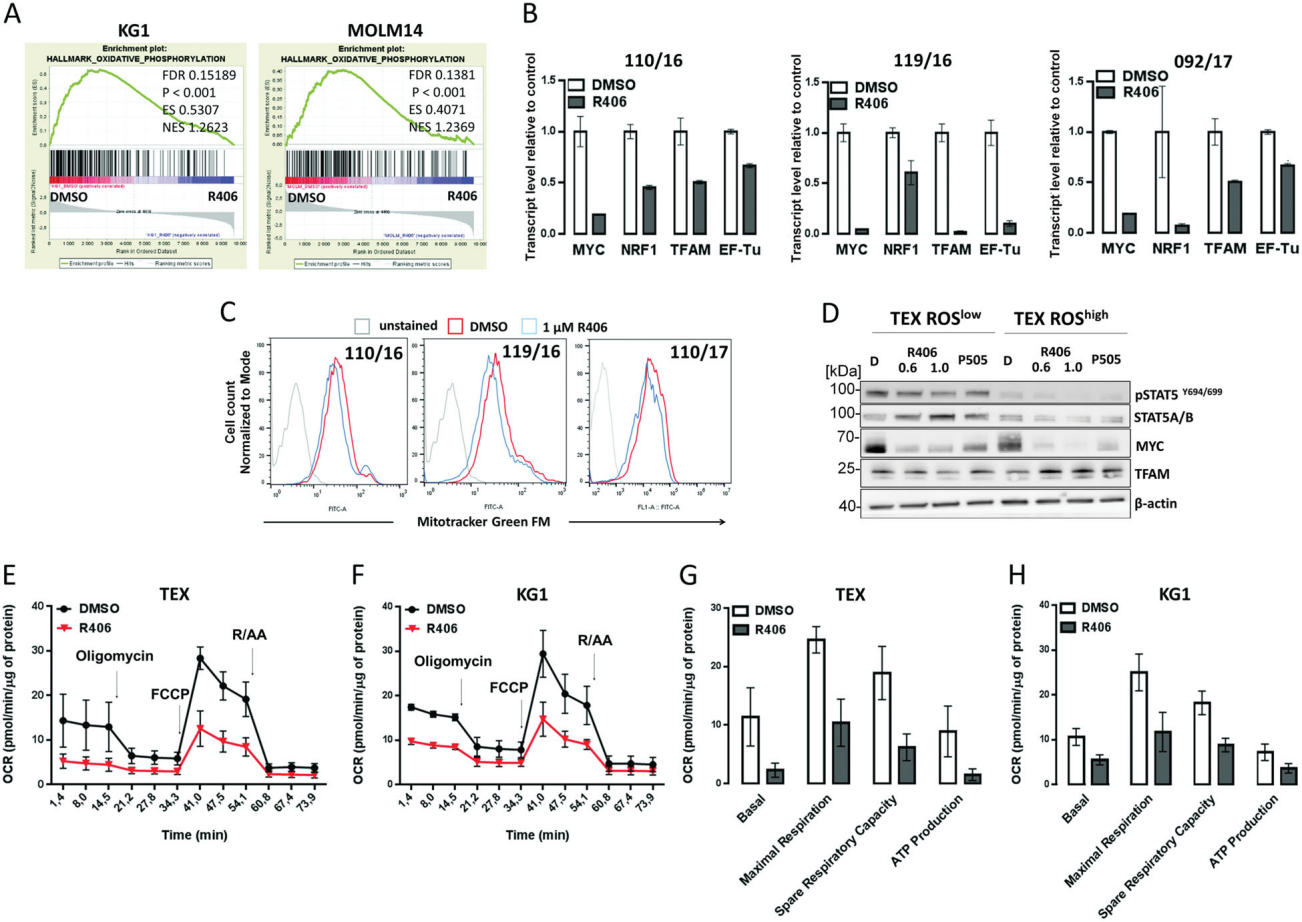
SYK inhibition decreases mitochondrial biogenesis and OXPHOS in acute myeloid leukemia (AML) cells. **A** GSEA plots showing downregulation of genes involved in oxidative phosphorylation in KG1 and MOLM14 cell lines after R406 treatment, in the same source dataset as in Fig. 4A and E. **B** The expression of MYC, NRF1, TFAM, and EF-Tu mRNA was quantified by qPCR using RNA isolated from primary AML blasts from three patients cultured in vitro for 24 h with DMSO or R406 (1 µM). 5S RNA was used as an internal standard. Bars represent mean + /− SD from four technical replicates. The results are shown relative to DMSO. **C** Changes of mitochondrial mass in AML cells treated with R406. Primary AML blasts from three patients, treated for 24 h with vehicle (red line) or 1 µM of R406 (blue line) were stained with Mitotracker Green FM, followed by flow-cytometry analysis. **D** Sorted TEX ROS-low and TEX ROS-high cells were treated for 24 h with vehicle or indicated doses of R406 (0.6 or 1 µM) or P505 (5 µM) as indicated. Thereafter, the phosphorylation status of ERK1/2, STAT5 and MYC and TFAM protein levels were assessed by immunoblotting. **E**, **F** Oxygen consumption rate (OCR) in TEX (1 × 10^5^/well) and KG1 cells (75 × 10^3^/well) after 24 h of treatment with R406 (0.6 and 0.4 μM, respectively) was evaluated by the Seahorse XF96 extracellular flux analyzer. Arrows indicate time points of the addition of oligomycin (1 µg/mL), FCCP (TEX 1.5 µM, KG1 1.25 µM), rotenone and antimycin A mix (R/AA, 1 µM each). Values shown are the average of three (KG1) or four (TEX) independent experiments + /− SEM normalized to the protein level. **G**, **H** Basal respiration, maximal respiration, spare respiratory capacity, and ATP production in TEX and KG1 cell treated as in **E** and **F**. Shown are average results of three (KG1) or four (TEX) independent experiments + /− SEM normalized to the protein level.

To gain insights into the mechanism of R406-mediated changes in clonogenic potential and LSCs function, we performed GSEA analysis of publicly available gene expression datasets from AML cell lines treated with either DMSO or R406^15^ and found that R406 significantly downregulated genes related to OXPHOS (Fig. 5A). We thus hypothesized that R406 treatment targets mitochondria and decreases OXPHOS metabolism in AML cells, contributing to the elimination of LSCs. To address this hypothesis, we assessed the differences in expression of mitochondria biogenesis genes (MYC, nuclear respiratory factor 1 (NRF1), transcription factor A mitochondrial (TFAM), and elongation factor thermo unstable (EF-Tu)), between ROS-low and ROS-high TEX subpopulations and found their higher expression in ROS-low TEX cells (Supplementary Fig. S5A). ROS-low TEX cells also had a higher expression of mitochondrially encoded mRNA for MT-ATP6 (a subunit of respiratory complex V) and MT-CYB (a subunit of respiratory complex III) (Supplementary Fig. S5A). Moreover, TEX ROS-low cells exhibited higher mitochondrial mass than TEX ROS-high cells (Supplementary Fig. S5B, C). Next, to assess whether SYK inhibition impairs mitochondria biogenesis, we incubated primary AML blasts and TEX, KG1 and MOLM14 with R406, and assessed NRF1, TFAM, EF-Tu, and MYC mRNA levels. R406 decreased the expression of these genes in all tested cells (Fig. 5B and Supplementary Fig. S5D). In line with changes in the mRNA expression, MYC and TFAM protein levels were markedly lower in ROS-low cells after incubation with R406 (Fig. 5D). Importantly, R406 also blocked STAT5 activity in ROS-low cells. When compared to ROS-low population, ROS-high cells expressed significantly lower levels of p-STAT5 and MYC that were further reduced by R406. However, in contrast to ROS-low cells, TFAM levels remained unchanged in ROS-high cells treated with R406 (Fig. 5D). Consistent with these findings, primary AML blasts and TEX cells treated with R406 exhibited decreased mitochondrial mass, as assessed by Mitotracker staining (Fig. 5C and Supplementary Fig. S5E).

To determine whether the observed changes have functional consequences, we assessed oxygen consumption rate (OCR) in KG1 and TEX cells treated 24 h with R406 or DMSO using Seahorse XF96 extracellular flux analyzer. SYK inhibition severely impaired OXPHOS metabolism in these cells, as indicated by reduced basal and maximal OCR, reduced spare respiratory capacity, and production of ATP (Fig. 5E–H). In contrast, SYK inhibition did not increase extracellular acidification rate (ECAR), indicating that these cells do not compensate OXPHOS block by switching their energy metabolism to glycolysis (Supplementary Fig. S5F). Of note, no increase in cell death was observed after 24 h of treatment with R406 (Supplementary Fig. S5J, K), indicating that decreased cell viability was not responsible for an observed overall reduction in metabolic activity. Taken together, these results demonstrate that SYK inhibition decreases mitochondrial biogenesis and oxidative metabolism in AML and highlight the new role of SYK in control of metabolic homeostasis of AML LSCs.

To ensure that the observed effects on mitochondrial biogenesis were mediated by SYK, we silenced its expression in TEX cells using RNAi. Having confirmed the SYK knockdown, we assessed the expression of genes involved in mitochondrial biogenesis and evaluated abundance of mitochondrially encoded respiratory chain genes. When compared to control cells, SYK-depleted cells exhibited markedly lower MYC, NRF1, TFAM, MT-ATP6, and MT-CYB abundance (Supplementary Fig. S5G, H), indicating that SYK knockdown phenocopies pharmacological inhibition of SYK in TEX cells. These results further confirm the role of SYK as an upstream modulator of the mitochondrial biogenesis in AML cells.

### SYK-dependent p-STAT5 activity increases MYC expression and OXPHOS metabolism in AML cells

**Fig. 6:**
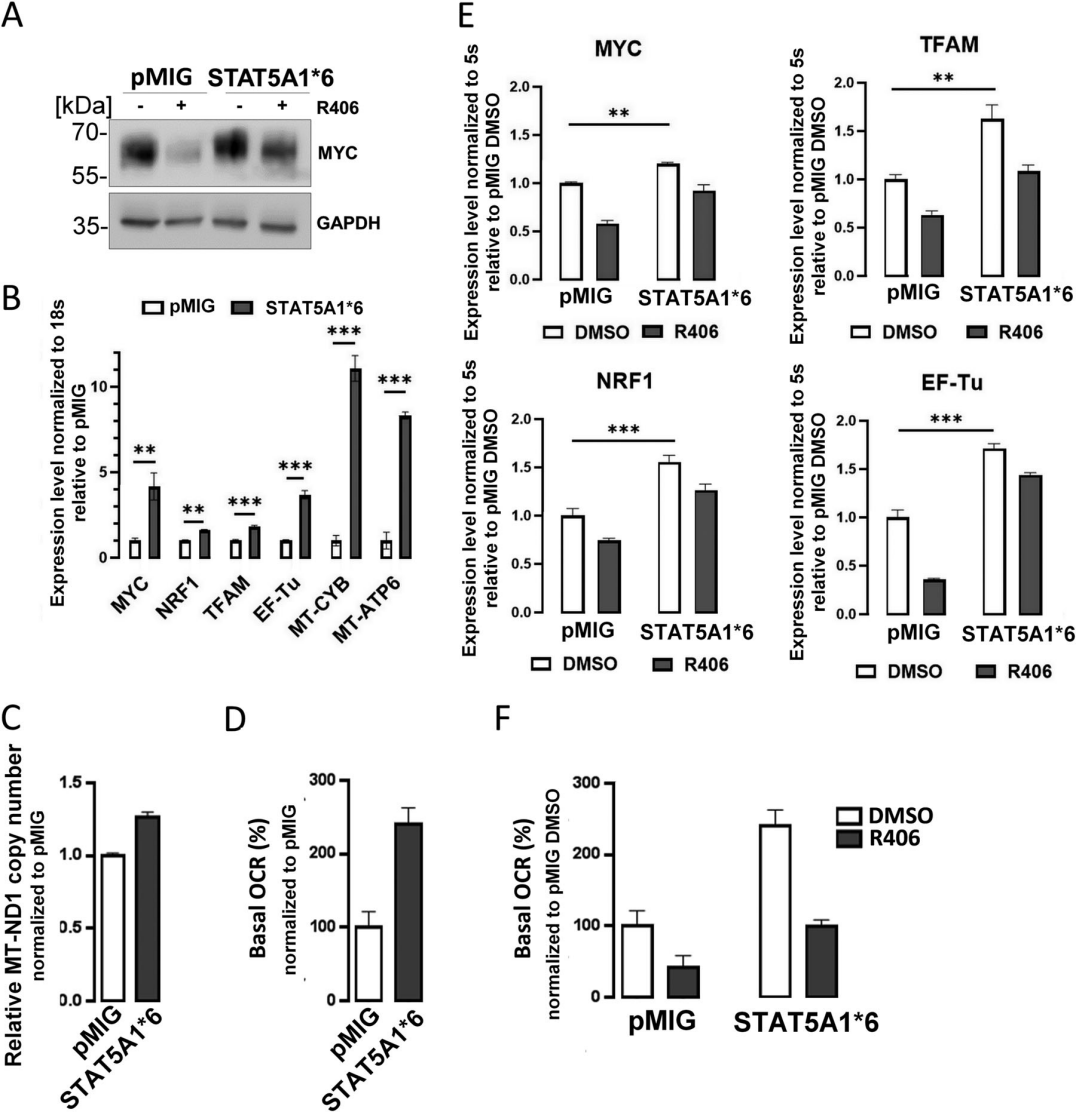
SYK-dependent p-STAT5 activity increases MYC expression, mitochondrial biogenesis, and OXPHOS. **A** KG1 cell were electroporated with either empty vector (pMIG) or a vector carrying constitutively active STAT5 mutant (STAT5A1*6). Forty-eight hours later, cells were treated with DMSO or R406 (0.4 µM) and lysed. Protein extracts were PAGE-separated and immunoblotted with anti-MYC antibody. **B** RNA was isolated from KG1 cells 48 h after electroporation with STAT5A1*6 or empty vector. The basal mRNA expression level of MYC, NRF1, TFAM, EF-Tu, MT-CYB, and MT-ATP6 was determined by qPCR using 18 s RNA as an internal standard. The experiment was repeated twice. Bars represent mean + /− SD from four technical replicates of a representative experiment. Data are presented relative to pMIG empty vector. **P < 0.01, ***P < 0.001. **C** Relative mitochondrial DNA copy number in KG1 cell transduced with either empty vector or STAT5A1*6. DNA was extracted from cells 48 h after electroporation, and qPCR was used to measure levels of mitochondrial ND1 gene relative to single-copy HGB gene. ND1/HGB ratio is shown relative to an empty vector containing cells. Bars represent mean + /− SD from four technical replicates. **D** Basal respiration in KG1 transduced with either STAT5A1*6 or empty vector, as evaluated by the Seahorse XF96 extracellular flux analyzer. Five replicate wells containing 75 × 10^3^ cells were analyzed. Mean values normalized to protein level +/− SD are plotted. Data are presented relative to empty vector control. **E** KG1 cells were electroporated with either STAT5A1*6 or empty vector and treated as described in **A**. Transcript levels of MYC, NRF1, TFAM, and EF-Tu were determined by qPCR using 5 s RNA as an internal standard. The experiment was repeated twice. Bars represent mean + /− SD from three technical replicates of a representative experiment. Data are presented relative to empty vector-transduced, DMSO-treated cells. **P < 0.01, ***P < 0.001 as determined by Student’s *t* test. **F** Cells were electroporated and treated as in **A**. Basal respiration was evaluated by the Seahorse XF96 extracellular flux analyzer. Bars represent means of four biological replicates + /− SD. Data are presented relative to empty vector, DMSO-treated control.

MYC is a STAT5 target gene and a positive regulator of mitochondrial biogenesis^35^. Given decreased abundance/activity of these proteins after SYK inhibition in ROS-low AML LSCs, we asked whether observed effects of R406 on mitochondrial respiration are dependent on STAT5A–MYC inhibition. To address this question, we used KG1 cells transduced with constitutively active STAT5A1*6 construct, in which SYK inhibition did not change STAT5 activity (Fig. 3A). We probed these cells for MYC abundance and found that STAT5A1*6-transduced cells had higher MYC level, when compared to control cells. However, SYK inhibition partially decreased MYC level in STAT5A1*6 cells, indicating that MYC expression is at least partially independent on STAT5 (Fig. 6A). Thereafter, we measured the expression of mitochondrial biogenesis genes in KG1 cells transduced with STAT5A1*6 or the empty vector, and found that active STAT5A increased expression of TFAM, NRF1, EF-Tu, and increased mitochondrial mass, as measured by the mitochondrial DNA copy number^36^ (Fig. 6B, C). STAT5A1*6-transduced cells also expressed higher mRNA levels of mitochondrial genome-encoded MT-ATP6 and MT-CYB. Most importantly, these cells exhibited increased OCR, indicating augmented OXPHOS (Fig. 6D).

We next determined whether R406 impact on mitochondrial biogenesis and OCR (presented in Fig. 5 and Supplementary Fig. S5) required the inhibition of STAT5 activity. For this purpose, we incubated STAT5A1*6 or empty vector-transduced KG1 cells with R406, and assessed the effects of this treatment on OXPHOS metabolism and expression of genes involved in mitochondrial biogenesis. In control cells, R406 treatment markedly reduced the mRNA and/or protein levels for MYC, TFAM, EF-Tu, and NRF1. Similar changes were observed in STAT5A1*6-expressing cells after incubation with R406, although the expression of these genes following SYK inhibition remained at significantly higher levels than in control cells (Fig. 6E). R406 reduced OCR both in control and STAT5A1*6-expressing cells, although in the latter, the OCR after R406 incubation also remained higher than in controls treated in the same manner (Fig. 6F). These findings are consistent with the partial inhibition of MYC in STAT5A1*6-expressing cells treated with R406 (Fig. 6A). Together, these results indicate that STAT5A activity in cells with active SYK is at least partially responsible for maintaining mitochondrial biogenesis and OXPHOS metabolism in AML cells, and its inhibition decreases OXPHOS. However, these results also highlight STAT5-independent mechanisms in this process.

### R406 sensitizes ROS-low AML cells to cytarabine

**Fig. 7:**
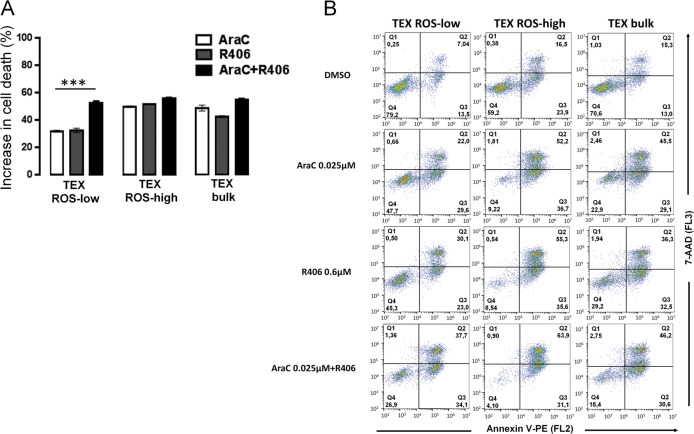
SYK inhibition sensitizes OXPHOS-dependent acute myeloid leukemia (AML) cells to AraC treatment. **A** ROS-low, ROS-high, and bulk TEX cells subsets were treated with DMSO, AraC (0.025 µM), R406 (0.6 µM), or combination of AraC and R406 for 72 h. Thereafter, cell death was assessed by annexin-V/PI staining, followed by flow-cytometry analysis. Bars represent mean of two experiments + /− SD. **B** Representative dotplots of cells treated as in panel **A**.

LSCs are thought to be resistant to conventional cytotoxic drugs used in standard AML treatment. As modulation of oxidative metabolism disorders LSCs function and characteristics, we hypothesized that SYK inhibition would sensitize OXPHOS-dependent, ROS-low cells to cytarabine (AraC). To test this hypothesis, we first assessed whether TEX ROS-low and TEX ROS-high populations have different sensitivity to AraC. As anticipated, we found that ROS-low TEX cells were more resistant to AraC treatment, as compared to TEX ROS-high cells (Fig. 7). When ROS-low cells were coincubated with R406, their sensitivity to AraC markedly increased. In contrast, co-treatment of TEX ROS-high cells with AraC and R406 did not further decrease the viability of these cells above the level induced by AraC alone. These observations indicate that R406-mediated targeting of OXPHOS metabolism in LSCs sensitizes them to AraC.

Although AraC remains the standard of care in first-line AML induction therapy, new targeted agents, such as BCL2-selective inhibitor venetoclax (ABT-199), were recently approved for AML treatment by EMA and FDA. Since venetoclax targets LSCs in a mechanism that involves OXPHOS inhibition^8,9^, we tested whether SYK blockade would synergize with venetoclax. In KG1, TEX and MOLM14 cells treated with ABT-199 and R406, cell viability decreased more robustly as compared to either drug used alone (Supplementary Fig. S6).

## Discussion

SYK is an important signaling hub in AML, activated by a number of upstream pathways. Consistent with the kinase’s oncogenic function, genetic or chemical SYK inhibition induces cell differentiation and death. However, although the inhibition of SYK activity is known to influence multiple downstream signaling pathways, their role(s) in mediating biological consequences of SYK blockade were incompletely understood. In this study, we demonstrate that SYK inhibition induces AML cells differentiation by modulation of the MEK/ERK1/2 pathway. ERK1/2 has been found to have anti- or pro-differentiating effects, depending on experimental conditions, type, intensity, and duration of the stimulation. In FLT3-ITD-positive AML cells, characterized by high SYK activity^14^, ERK1/2 phosphorylates and inactivates the C/EBPα, a transcription factor involved in granulocytic differentiation^23,37^. Using the gain-of-function MEK kinase mutant, which constitutively activates ERK1/2, we demonstrate that SYK-inhibitor-induced differentiation requires blocked MEK/ERK1/2 activity. According to this signaling framework, inhibition of ERK1/2 might exhibit the pro-differentiating effect at least partially through C/EBPα, but given ERK1/2 broad substrate specificity, connections with other pathways cannot be excluded. In a study by Carnevale et al., the differentiation-inducing effect of SYK inhibitor was associated with suppression of mTOR, and simultaneous inhibition of 4E-BP1 and SYK induced cell differentiation more efficiently than either agent alone^18^. Given the cross-talk between MEK/ERK1/2 and mTOR pathways, these observations might in fact have a common explanation. As ERK1/2 phosphorylates p90RSK kinase, which activates mTOR^38–40^, the inhibition of ERK1/2 might also lead to a decreased mTOR activity. Although our studies strongly implicate the MEK/ERK1/2 pathway in SYK-inhibitor-induced differentiation, this cascade likely plays a broader role in mediating responses to SYK inhibitor. By using an integrated proteogenomic approach, Cremer et al.^41^ demonstrated that innate or acquired activation of the RAS/MAPK/ERK1/2 signaling pathway confers resistance to SYK inhibition, indicating that this pathway is a major effector and reciprocal modulator of SYK-dependent signaling in AML.

In addition to the induction of differentiation, we report that SYK inhibition also reduced CD34^+^CD38^−^CD123^+^ and CD34^+^CD38^−^CD25^+^ compartments, and decreased the viability of LSC-enriched ROS-low population. In line with these observations, primary leukemic blasts treated ex vivo with R406 exhibited lower engraftment potential in immunocompromised NSG/J mice. Mechanistically, these effects are mediated by SYK-inhibitor-triggered changes in control of mitochondrial biogenesis and metabolic function in LSCs. This self-renewing cell population is highly susceptible to OXPHOS inhibition, compared to bulk leukemic population^10^. Consistent with the dependence on OXPHOS, LSCs have higher mitochondrial mass and distinct morphology (this study and ref. ^42^). These observations highlight the essential role of mitochondria for LSCs survival and indicate that LSCs metabolic characteristics can be exploited therapeutically.

The differences in mitochondrial mass/function in LSCs appear to be at least partially dependent on STAT5 and its target genes, including MYC, a well-defined inducer of mitochondrial biogenesis^35^. In AML cell lines with enforced expression of constitutively active STAT5, we observed increased mRNA levels of factors regulating mitochondrial biogenesis, including NRF1, TFAM, EF-Tu, and MYC. Of note, shRNA-mediated inhibition of TFAM in AML was sufficient to decrease OCR and OXPHOS in AML cells^43^. Consistent with a putative STAT5 role in the regulation of mitochondrial genome function^44^, we also observed increased expression of mitochondrial genome-encoded electron transport complexes (ETC) (MT-ATP6 and MT-CYB) in cells with active STAT5. Taken together, our findings suggest that the SYK signaling pathway in LSCs participates in their metabolic reprogramming in a mechanism that at least partially involves STAT5.

Recent studies indicate that LSCs differ from the “bulk” population also in utilizing amino acids to fuel tricarboxylic acid cycle^9^. Decreased availability or pharmacological inhibition of cellular import and metabolism of amino acids with venetoclax and azacytidine impairs LSCs survival, and produces deep and durable clinical remissions^8,9^. Since MYC is a potent inducer of amino acid transporters SLC7A5 and SLC1A^45^, and given its decreased abundance in LSCs following SYK inhibition, it is tempting to speculate that SYK inhibition-mediated decrease in OCR/OXPHOS in LSCs might involve impaired cellular amino acid import. Since MYC is partially independent of STAT5 in LSCs, these observations would explain only partial rescue effect mediated by the introduction of constitutively active STAT5 on mitochondrial biogenesis and OXPHOS.

In addition to the direct effects of SYK inhibition on cell differentiation, metabolism, and survival, SYK inhibition in LSCs increases their sensitivity to AraC. Since LSCs can acquire resistance to either AraC or R406, this observation suggests that the combination therapy would be more likely to eradicate LSCs and prevent the emergence of cells resistant to AraC or SYK inhibitor. Residual leukemic cells that remain after AraC therapy rewire their metabolism and boost OXPHOS^46^. Similarly, cells resistant to the LSC-targeting therapy with venetoclax and azacytidine^9^, reprogram their bioenergetics, become independent of amino acid-derived carbons, and utilize palmitate instead. These observations highlight the unique metabolic plasticity of AML LSCs that limit therapeutic options available upon acquisition of resistance, underscoring the need to effectively target LSCs in the first line of treatment. Sensitization of ROS-low cells to AraC following SYK inhibition provides a starting point for further preclinical and clinical development of such combination therapy. Importantly, published data demonstrate that SYK activity may be dispensable for normal hematopoiesis^11,15^, suggesting that there is a therapeutic index for R406 in AML. In fact, phase II clinical trials testing R406 for the treatment of idiopathic thrombocytopenic purpura and rheumatoid arthritis did not report significant neutropenia among the treatment groups^21,47,48^.

In conclusion, our study demonstrates that SYK inhibition in AML favors cell differentiation in MEK-ERK1/2 dependent manner, targets LSCs through modulation of OXPHOS metabolism and increases their sensitivity to cytarabine. As SYK is expressed in a majority of AML cells, our study supports future clinical trials testing the combination of SYK inhibitors with standard chemotherapeutics.

## Materials and methods

### Primary AML blasts and cell lines

Leukemic samples from bone marrow were obtained after informed consent from 17 AML patients at diagnosis or relapse. The collection and use of human tissue for this study were approved by local Bioethics Committee Board based on The Declaration of Helsinki. Mononuclear cells were isolated from primary samples using density gradient centrifugation (Ficoll-Hypaque; density 1.077 g/mL, Sigma-Aldrich) within 24 h from harvesting. Cells were then either cryopreserved in liquid nitrogen in the presence of 90% fetal bovine serum (FBS) (Biovest) and 10% DMSO, or cultured at a density 1 × 10^6^/mL in S7 medium (83% IMDM, 15% BIT, 500 nM SR1, 500 nM UM729 (all from StemCell Technologies), 100 ng/ml SCF, 20 ng/ml G-CSF, 50 ng/ml FLT3-L, 20 ng/ml IL-3 (R&D), 0.1 mM β-Mercaptoethanol, 1% penicillin/streptomycin, 1% GlutaMAX 1% (Lonza))^49^, or RPMI-1640 medium (Lonza) containing 10% heat-inactivated FBS, 2mM L-glutamine, 10 mM HEPES, and 100 U/mL of penicillin/streptomycin, at a density 1 × 10^6^/mL.

AML cells lines (KG1, MOLM14, HEL, KASUMI1, MOLM16, U937, THP-1, OCI-AML3) were obtained from DSMZ (Braunschweig, Germany) and cultured according to DSMZ instructions. TEX leukemia AML cell line was kindly provided by Prof. John E. Dick (University Health Network (UHN), University of Toronto, Toronto, Ontario, Canada)^34^. TEX cells were maintained in IMDM (Iscove’s modified Dulbecco’s medium), 15% FBS (Sigma), 2 mM L-glutamine, 1% penicillin–streptomycin (Lonza), 20 ng/mL SCF (stem-cell factor), 2 ng/mL IL-3 (all from R&D). 293T cells were cultured in DMEM medium (Gibco) supplemented with 10% FBS and 100 U/ml of penicillin/streptomycin (Lonza). All cells were kept in a humidified incubator in 5% CO_2_ at 37 °C.

### Chemicals

The small-molecule SYK inhibitors, R406, entospletinib (GS-9973), PRT062607 (P505) and Venetoclax (ABT-199) from Selleckchem, were dissolved in DMSO at 10 mM, and stored at −80 °C. Compounds were used at indicated final concentrations. DMSO was used as a control, added in equivalent volumes not exceeding 0.1%. Phorbol 12-myristate 13-acetate (PMA), oligomycin, FCCP, rotenone, and antimycin A (Sigma-Aldrich) were dissolved in DMSO and stored at –20 °C.

### Cell proliferation, cell death, and clonogenicity assays

Cells were plated in triplicates at 25,000–35,000 cells/well in 100 μl of appropriate growth medium in a 96-well plate and cultured in the presence of indicated concentrations of R406 or P505. After 72 h, cell proliferation was assessed using MTS assay (CellTiter 96 Aqueous Non-Radioactive Cell Proliferation Assay kit, Promega) according to the manufacturer’s instructions. The absorbance at 490 nm was determined using a microplate reader (Multiscan GO, Thermo Scientific). For assessment of cell death, cells were stained with Annexin-V and/or propidium iodide (PI) (Annexin V apoptosis detection kit I; BD Pharmingen) according to the manufacturer’s instructions, and analyzed with flow cytometry and FlowJo software (FlowJo, LLC). To assess the clonogenic potential, KG1, MOLM14 (5 × 10^3^), and TEX cells (35 × 10^3^) were plated in triplicates in GFH4434 medium (StemCell Technologies) containing either DMSO or R406 at the indicated concentration for 14 days; thereafter, the colonies were counted under the light microscope. For replating, cells were pooled from duplicate plates, washed twice with PBS, and 5 × 10^3^ cells were replated in GFH4434 medium. After 14 days, colonies were counted under the light microscope.

### Quantitative nitroblue tetrazolium reduction assay (qNBT assay)

The ability of AML cells to produce O_2_^−^ in response to differentiation stimuli was assessed in an NBT reduction assay as described previously^50^. Briefly, after 5 days of R406 or DMSO treatment (*n* = 2), AML cells (1 × 10^5^) were mixed (1:1) with PBS solution containing 1 mg/mL NBT (Sigma-Aldrich) and 4 µg/mL PMA (Sigma-Aldrich). After 2 h of incubation, the cells were washed twice with PBS, fixed with methanol, and air‐dried. Then, 120 µL of 2 M NaOH was added to the cells to solubilize cell membranes, and subsequently 140 µL of DMSO was added to dissolve formazan deposits. After 10 min of shaking (room temperature), the dissolved NBT solution was transferred to a 96‐well plate, and the absorbance at 620 nm was read on a microplate reader (Multiscan GO).

### Western blot analysis

Western blot analysis was performed essentially as described previously^25^. Antibodies used in the study are listed in Supplementary Table S1.

### Vectors

pBabe-puro-MEK-DD (encoding MEK with phosphomimetic activating mutations), pBabe-puro and pMIG were obtained from Addgene (plasmid #15268^51^, #1764^52^, and #9044, respectively). The pMIP plasmid was obtained from pMIG trough replacement of GFP marker with puromycin resistance gene. The cDNA encoding constitutively active STAT5A form (STAT5A1*6)^30^ was synthesized by ATG:biosynthetics, (Merzhausen, Germany) and inserted into the *BglII* and *XhoI* sites of the pMIG and pMIP vectors.

### Retroviral transfections

VSV-G pseudotyped retrovirus was produced by transfecting HEK 293T cells with 10 µg of either pBABE-puro-MEK-DD, pMIP-STAT5A1*6, or appropriate empty controls, together with the envelope (VSV-G, 5 µg) and the packaging (pKAT, 10 µg) plasmids, using Lipofectamine 2000 transfection reagent (Life Technologies) as previously described^53^. After 24 h, supernatants were harvested, filtered (0.42 µM), mixed with polybrene (8 µg/ml) and used to infect KG1 and MOLM14 cell lines. Seventy-two hours after infections, cells were subjected to puromycin selection (0.5 and 0.7 μg/mL, respectively).

### Electroporation

Electroporation of plasmid DNA into KG1 cells was performed using Neon Transfection System MPK5000 (Invitrogen) and 100 µL NeonTip. Cells were plated in a complete medium at 0.6 × 10^6^/mL the day before electroporation. KG1 cells (5 × 10^6^) were electroporated with 10 µg of pMIG or pMIG-STAT51*6 plasmid (1700 V, 20 ms, 1 impulse).

### Quantitative real-time polymerase chain reaction (qPCR)

The total RNA was isolated from AML cells using GeneMATRIX Universal RNA purification kit (EURx). In all, 1 μg of RNA was reverse-transcribed with Transcriptor First-Strand cDNA synthesis kit (Roche). The qPCR reactions were performed in triplicate with SYBR Green Master MIX (Applied Biosystems) and gene-specific primers (primers sequence and details are described in Supplementary Table S2) using CFX96 qPCR system (Bio-Rad). C_T_ values were normalized to housekeeping genes: GAPDH (for differentiation-associated gene studies) or 18 s and 5 s (for expression of mitochondrial biogenesis genes). A relative transcript abundance was quantified using the ΔΔC_T_ method.

Mitochondrial DNA was quantified relative to the nuclear DNA using two independent PCRs and two pairs of primers. The first PCR amplified the NADH dehydrogenase-1 (*ND1)* gene in mtDNA, while the second PCR was used to amplify single-copy nuclear human globulin (*HGB*) gene (Supplementary Table S2 and Supplemental Methods).

### Immunophenotyping, mitotracker staining, and flow cytometry

Cells were washed once with PBS and stained for 30 min at RT in the dark with fluorochrome-conjugated antibodies diluted as recommended by the manufacturer (Supplementary Table S3). Activity of SYK in primary AML blasts and AML cell lines were assessed in 1 × 10^6^ cells using intracellular phospho-specific flow cytometry (Phosflow Protocol II for Human PBMCs, BD Biosciences)^54^. To determine mitochondrial mass, cells were incubated in PBS containing 50 nM of Mitotracker Green FM (Thermo Scientific) for 30 min at 37 °C and washed with PBS. All FACS data were acquired on CytoFLEX (Beckman Coulter) flow cytometer and analyzed using FlowJo software (FlowJo, LLC).

### Cell sorting based on endogenous ROS levels

In total, 50 × 10^6^ cells were stained with the redox-sensitive probe CM-H_2_DCFDA (5 µM) in PBS buffer for 15 min at 37 °C in the dark. Cells were then washed with PBS, resuspended in complete media, and incubated for 30 min at 37 °C in the dark. Thereafter, AML cells with bottom 15% or top 15% of dye fluorescence distribution (ROS-low and ROS-high cells, respectively) were collected using a BD ARIA II cell sorter (BD Biosciences).

### Primary AML blasts ex vivo treatment and xenotransplantations studies

All animal experiments described in this study was performed in accordance with and approved by the 2nd Local Ethical Committee for Animal Research in Warsaw, Poland. PDX model was developed using primary AML bone marrow blasts from a single patient (patient 096/17). Primary cells were injected via tail vein into sublethally irradiated (2.15 Gy) female 3-month-old NOD.Cg-Prkdc^SCID^Il2rg^tm1Wjl^/J (NSG/J) mice and after the development of overt leukemia, mice were euthanized and bone marrow cells (BMCs) were harvested. Following erythrocyte lysis (BD Pharm Lyse Buffer), BMCs were cultured for 24 h in S7 medium containing either DMSO or R406 (4 µM). After incubation, cells were washed with PBS, and 1.75 × 10^6^ of viable cells were resuspended in 350 μl ice-cold PBS and re-transplanted into female NSG/J mice via tail vein injection (*n* = 5 per group, no randomization). Eight weeks after the transplantation, mice were killed and the engraftment of human CD45^+^CD33^+^CD34^+^ cells in mouse bone marrow was assessed by FACS in a blinded fashion.

### Oxygen consumption rate (OCR) assessment

OCR was assessed using a Seahorse XF96 flux analyzer (Seahorse Bioscience). KG1 (75 × 10^3^ cells/well) and TEX (1 × 10^5^ cells/well) cells were cultured in a 96-well XF96 culture plate (Seahorse Bioscience) coated with BD Cell-Tak (BD Biosciences) in their regular growth medium for 24 h with or without R406. An hour prior to the analysis, cells were washed with PBS and resuspended in XF base medium (pH 7.4) (Agilent Technologies) supplemented with glucose (5 mM), L-glutamine (1.6 mM), and pyruvate (1 mM). Plates were incubated for 1 h at 37 °C in a CO_2_-free incubator and transferred to the XF96 analyzer. OCR was measured at baseline and after the injection of oligomycin (1 µg/ml), FCCP (1.5 µM for TEX and 1.25 µM for KG1), rotenone/antimycin A (1 µM). After measurements, cells were washed with PBS, lysed in 10 μL of 0.1% Triton/PBS solution and frozen at −80 °C overnight. Thereafter, the protein concentration in each well was measured using BCA (Copper(II) sulfate solution and Bicinchoninic Acid solution, Sigma-Aldrich). Data were normalized to protein concentration and analyzed using Wave 2.0 software (Agilent Technologies).

### Gene set enrichment analysis (GSEA) and statistical analyses

In silico data mining was performed on a gene expression dataset obtained from MOLM14 and KG1 cells grown in 4 µM R406 for 24 h, deposited under Gene Expression Omnibus ID GSE46302. GSEA was performed as previously described^25^. All continuous data are expressed as means, with standard deviations (SD). Shapiro–Wilk test was used to determine normal distribution. Statistical analyses were performed using two-sided, unpaired Student’s *t* test. When indicated, paired *t* test was used. Differences were considered statistically significant at *P* < 0.05. Sample size (*n*) and the number of replicates are provided in corresponding figure legends.

## Supplementary information

Supplementary material

Supplementary Figure 1

Supplementary Figure 2

Supplementary Figure 3

Supplementary Figure 4

Supplementary Figure 5

Supplementary Figure 6
