# Supplementary material for: SYK inhibition targets acute myeloid leukemia stem cells by blocking their oxidative metabolism
Source: Cell Death Dis. 2020 Nov 6;11(11):956. doi: 10.1038/s41419-020-03156-8 (PMC7648638; doi:10.1038/s41419-020-03156-8)
Supplement: Supplementary file 1 — Supplementary material [file 41419_2020_3156_MOESM1_ESM.docx]

**Supplemental information**

Anna Polak^1^, Emilia Bialopiotrowicz^1^, Beata Krzymieniewska^2^, Jolanta Wozniak^2^, Marta Stojak^3^, Magdalena Cybulska^4^, Ewelina Kaniuga^4^, Michał Mikula^4^, Ewa Jablonska^1^, Patryk Gorniak^1^, Monika Noyszewska-Kania^1^, Maciej Szydlowski^1^, Karolina Piechna^1^, Katarzyna Piwocka^5^, Lukasz Bugajski^5^, Ewa Lech-Maranda^6^, Joanna Barankiewicz^6^, Agnieszka Kolkowska-Lesniak^6^, Elzbieta Patkowska^6^, Eliza Glodkowska-Mrowka^1^, Natalia Baran^7^, and Przemyslaw Juszczynski^1*^

*1 Department of Experimental Hematology, Institute of Hematology and Transfusion Medicine, Warsaw, Poland*

*2 Department of Diagnostic Hematology, Institute of Hematology and Transfusion Medicine, Warsaw, Poland*

*3 Jagiellonian Centre for Experimental Therapeutics (JCET), Jagiellonian University, Kraków, Poland*

*4 Department of Genetics, Maria Sklodowska-Curie National Research Institute of*Oncology,*Warsaw, Poland*

*5 Laboratory of Cytometry, Nencki Institute of Experimental Biology, Polish Academy of Sciences, Warsaw, Poland*

*6 Department of Hematology, Institute of Hematology and Transfusion Medicine, Warsaw, Poland*

*7. Department of Leukemia, The University of Texas MD Anderson Cancer Center, Houston, TX, USA*

**Supplemental methods**

**Mitochondrial genome copy number analysis using PCR**

Genomic DNA was isolated from AML cells using AllPrep DNA*/*RNA Mini Kit (Qiagen). Samples were assessed in triplicates in a 96 well plate with CFX96 Touch Real-Time PCR Detection System (Bio-Rad). The reactions were performed in a total volume of 14µl including 1× SYBR Green Mastermix (Applied Biosystems), 215nM ND1-F (or HGB-1) primer, 215nM ND1-R (or HGB-2) primer and 4ng of total DNA. The amplification condition were as follows: for the *ND1* gene: 95°C for 10 min., followed by 40 cycles of 15 s at 95°C and 1min. at 60°C. For the HGB gene: 95°C for 10 min, followed by 40 cycles of 15 s at 95°C and 1min. at 56°C. The PCRs for *ND1* and HGB were performed on separate 96 well plates with the same samples in the same well positions. The relative mtDNA copy number was calculated by classical ΔΔCt method. ΔCt was calculated using the following formula: ΔCt = Ct(mtDNA gene)−Ct(nDNA gene). ΔΔCt was calculated as 2^−ΔCt^.

**May-Grünwald-Giemsa stain**

For morphological assessment of differentiation, AML cells were incubated for 5 days with R406 at the indicated concentrations. Thereafter, 4×10^6^ cells were smeared onto a microscope slides, fixed with methanol and stained for 3 min. with May-Grünwald, followed by 13 min staining with 25% Giemsa solution (Merck). Stained smears were then analyzed using light microscope (Olympus CX41) equipped with digital camera SC30, under 100x objective lens with oil immersion. Images were captured using CS-EN-V1 cell Sens Entry software*.*

**siRNA knockdown**

SYK-targeting (E-003176-00-0005) or control (E-003176-00-0005) Accell Human siRNA were obtained from Dharmacon (Thermo Scientific), and delivered to TEX cells according to manufacturer recommendations. Briefly, on the day of transfection, 10^6^ cells were resuspended in Accel siRNA delivery media (Dharmacon) supplemented with 0.5% FBS (Biovest) at concentration 0.6×10^6/^mL, and mixed with 1µM of siRNA. After 72h-96h of incubation, cells were pelleted and assessed for knockdown efficiency on a protein level using western blotting.

**Cell cycle analysis**

AML cells were washed with PBS, fixed overnight at -20°C in 70% ethanol, washed twice with PBS and resuspended in 500 μL of PI staining solution (PBS, 4mM sodium citrate, 10μg/ml RNAseA and 50μg/ml PI). Flow cytometry was performed with a CytoFLEX flow cytometer (Beckman Coulter), and the DNA histograms were further analyzed using FlowJo software (FlowJo, LLC).

**Ki-67 staining**

Cells were stained using Alexa Fluor 647-conjugated anti-Human Ki-67 antibody (BD) according to manufacturer instructions. Data were acquired using CytoFLEX flow cytometer (Beckman Coulter), and analyzed using FlowJo software (FlowJo, LLC).

**Secondary transplants**

Bone marrow cells from mice engrafted with R406 and DMSO treated leukemias were harvested, frozen and stored in liquid nitrogen. For secondary transplant, cells were thawed and 1.75×10^6^ cells resuspended in 350uL of PBS were reinjected into recipient NSG/J mice for reexpansion (n=4 per group). 8 weeks after re-transplantation, AML engraftment was assessed by FACS analysis for the presence of hCD45+ cells in mouse peripheral blood.

**Table S1. Antibodies used in the study**

| Name | Company | Cat No. |
| --- | --- | --- |
| p-ERK ^(Y202/T204)^ | Cell Signaling | 4370 |
| ERK | Millipore | 05-1152 |
| p-STAT5 ^(Y694)^ | Cell Signaling | 9359 |
| STAT5 | Cell Signaling | 9363 |
| MYC | Abcam | Ab320072 |
| TFAM | Sigma | SAB1401383 |
| GAPDH | Millipore | MAB374 |
| SYK | Santa Cruz | SC1240 |
| Anti-Mouse IgG–Peroxidase antibody | Sigma | A9044 |
| Anti-Rabbit IgG −Peroxidase antibody | Sigma | A0545 |

**Table S2. Sequences of primers used in the study**

| Name | Sequence 5’-3’ |
| --- | --- |
| PU1_F | AGTCTTGGCCACCAGGTC |
| PU1_R | ATCCGCCTCGCCGTCAGA |
| CEBP/β_F | ATCGACTTCAGCCCGTACC |
| CEBP/B_R | AAGTCGTGGTGCTGCCCG |
| GFI1_F | ATCGCAGCTGACCGAAGC |
| GFI1_R | CATTGACTTCTCCGAGGCTGG |
| IRF8_F | AGACGAGGTTACGCTGTGC |
| IRF8_R | TGCCACGCCTAGTTTGCATT |
| M-CSF1R_F | CCAGAGAGCATCTTTGACTGTG |
| M-CSF1R_R | GGGTAGGGATTCAGCCCAAG |
| ND1-F | CCCTAAAACCCGCCACATCT |
| ND1-R | GAGCGATGGTGAGAGCTAAGGT |
| HGB-1-F | GTGCACCTGACTCCTGAGGAGA |
| HGB-2-R | CCTTGATACCAACCTGCCCAG |
| MT-CYB_F | ACCCCCTAGGAATCACCTCC |
| MT-CYB_R | GCCTAGGAGGTCTGGTGAGA |
| MT-ATP6_F | GCGCCACCCTAGCAATATCA |
| MT-ATP6_R | AGGCTTGGATTAAGGCGACA |
| 18SF | AGGAATTGACGGAAGGGCAC |
| 18SR | GGACATCTAAGGGCATCACA |
| NRF1_F | CAGCACCTTTGGAGAATGTGGTG |
| NRF1_R | AGGCGGCAGTTCTGAGTTAACCT |
| TFAM_F | ACTGCGCTCCCCCTTCAGTTT |
| TFAM_R | CCTCCAACGCTGGGCAATTCT |
| EF-TU_F | GCTACGACGAGATCGTCAAGGA |
| EF-TU_R | GCCCTTGAACCACGGCATGTT |
| MYC_F | TCTCTCCGTCCTCGGATTCT |
| \| **MYC_R** \| \| --- \| | TTGTTCCTCCTCAGAGTCGCT |
| GAPDH_F | AGCCTCCCGCTTCGCTCTCT |
| GAPDH_R | CGACCAAATCCGTTGACTCCGACC |
| 5S_F | TCTCGTCTGATCTCGGAAGC |
| 5S_R | AGCCTACAGCACCCGGTATT |

**Table S3. Antibodies used in flow cytometry experiments**

| Name | Company | Cat. No |
| --- | --- | --- |
| Zap-70(pY319)/Syk(pY352) PE | BD Biosciences | 557881 |
| PE Mouse IgG1, κ Isotype Control | BD Biosciences | 551436 |
| PE Mouse Anti-Human CD123 | BD Biosciences | 555644 |
| Monoclonal Mouse Anti-Human CD25, Interleukin-2 Receptor/RPE, Clone ACT-1 | Dako | R0811 |
| APC anti-human CD34 Antibody Clone 8G12 | BD Biosciences | 345804 |
| FITC anti-human CD38 Antibody Clone HB7 | BD Biosciences | 340909 |
| PE anti-human CD34 Antibody Clone 8G12 | BD Biosciences | 345802 |
| CD45 FITC  Clone 2D1 | BD Biosciences | 345808 |
| APC anti-human CD33 Antibody Clone P67.7 | BD Biosciences | 345800 |
| Mouse IgG1 PE  Clone X40 | BD Biosciences | 345816 |
| Mouse IgG1 FITC  Clone X40 | BD Biosciences | 345815 |
| Mouse IgG1 APC  Clone X40 | BD Biosciences | 345818 |
| Alexa Fluor 647 mouse anti-Ki67  Clone B56 | BD Biosciences | 558615 |
| PE Mouse Anti-human CD14Clone MφP9 | BD Biosciences | 347497 |

**Table S4. Characteristics of AML patients who provided leukemia specimens used** **in the** **study** (symbols used to describe the phenotype: (+), >75% expression on blasts; (+/-), 50-75% expression on blasts; (-/+), 20-50% expression on blasts; (-), <20% expression on blasts)

| Specimen | FAB/Clinical Status | Gender/  Age (years) | Genetic abberations | Phenotype | Tissue source | Blasts | |
| --- | --- | --- | --- | --- | --- | --- | --- |
| 01/14 | AML M4 | Male/61 | BAALC overexpression (+) | Myeloblasts: CD34+CD117+DR+CD33-/+ CD13+/-CD15+CD38+  Monocytoid cells: CD64+CD14-/+CD36+CD33+CD15+CD11b+DR+CD38+/-CD117-/+CD34-/+ | BM | | Myeloblasts 43%  Monocytoid cells 30% |
| 02/14 | AML M2 | Female/29 | FLT3-ITD (+)  MLL-PTD (+)  t(8;21) (-)  t(4;11) (-)  inv(16) (-) | CD34+CD117+CD13+CD33+CD15-DR-/+CD38+CD7-MPO+/- | BM | 72% | |
| 03/13 | AML M2 | Male/76 | FLT3-ITD (-) | CD34+CD117+CD13+CD33-/+DR+CD38+CD7+MPO- | BM | 70% | |
| 04/15 | AML M0 | Female/70 | Del (5q) | CD34+CD117+CD13+CD33+CD38+/-DR+CD123++ | BM | 37% | |
| 05/13 | AML M2 | Female/45 | FLT3-ITD (+)  MLL-PTD (+)  8;21) (-)  t(4;11) (-)  inv(16) (-) | CD117+CD34-CD13+ CD33+/-CD15-DR+CD38-/+MPO+ | BM | 57% | |
| 06/16 | AML M2 from MDS | Female/80 | Del (5q) | CD34+CD117+CD33+CD13+DR+CD38-/+ | BM | 30% | |
| 096/17 | AML M1/ de novo AML | Female/75 | BAALC overexpression (+)  t(8;21) (–)  inv(16) (–)  FLT3-ITD (–)  KMT2A-PTD (–) | CD34+ CD117+ CD13+ CD33+ CD15+/- HLA-DR+dim, CD38+dim CD7+/- CD56- MPO- | BM | 90% | |
| 110/17 | AML M1/ de novo AML | Female/86 | BAALC overexpression (–)  FLT3-ITD (+)  KMT2A-PTD (+)  t(8;21) (–)  NPM1 (–) | CD34+CD117-/+ CD13+CD33- CD15-/+  HLA-DR- CD7+CD56-MPO-/+ | BM | 93% | |
| 110/16 | AML M2/ de novo AML | Female/70 | FLT3-ITD (–)  KMT2A-PTD (–)  Inv (16) (–)  t(8;21) (–) | CD34+CD117+ CD13+CD33+ CD15- HLA-DR+ CD38+CD7+ CD56-/+CD2+/-MPO- | BM | 81% | |
| 088/17 | AML M1/ de novo AML | Male/24 | BAALC overexpression (+)  FLT3-ITD (–)  t(8;21) (–)  NPM1 (–)  Inv(16) (–) | CD34+CD117-/+ CD13-/+dim CD33+dimCD15-  HLA-DR+ CD38+dim CD7+CD56-MPO- | BM | 92% | |
| 070/18 | AML M1/ de novo AML | Female/33 | BALLC overexpression (+)  FLT3-ITD (–)  t(8;21) (–)  Inv(16) (–)  BCR/ABL (–) | CD34+CD117+ CD13- CD33+CD15-  HLA-DR+dim  CD38-/+dim CD7+CD56-MPO- | BM | 87% | |
| 043/17 | AML M4/ de novo AML | Female/79 | FLT3-ITD (+)  inv(16) (–)  t(8;21) (–) | CD13+CD33+CD34+/-CD117+HLA-DR-CD123++ | BM | 61% | |
| 017/18 | AML NOS | Female/59 | FLT3-ITD (+)  t(8;21) (–)  inv(16) (–)  FLT3-TKD (–) | CD117+CD34+HLA-DR+CD64-/+^dim^CD13+CD33+CD36-CD15+CD11c- CD56-CD7+CD2 | BM | 74% | |
| 017/17 | AML M2/ de novo AML | Male/55 | BAALC overexpression (+)  inv(16) (–)  t(8;21) (–)  FLT3-ITD (–)  KMT2A-PTD (–) | CD34+CD117+ CD13+CD33+dim CD15- HLA-DR+ CD38-CD7-CD56-MPO-/+ | BM | 66% | |
| 010/18 | AML M2/ de novo AML | Female/39 | FLT3-ITD (+)  Inv(16) (+)  BAALC overexpression (+)  KMT2A-PTD (–)  NPM1 (–)  Mutacje CEBPA (–)  t(8;21) (–) | CD34+CD117+ CD13+ CD33+ CD15+ HLA-DR+  CD38-/+dimCD7-CD56- | BM | 50% | |
| 119/16 | AML NOS/  Relapse | Female/45 | BAALC overexpression (+)  FLT3-ITD (–)  inv(16) (–)  t(8;21) (–)  KMT2A-PTD (–) | CD34+CD117+ CD13+CD33+/-CD15- HLA-DR+CD38-/+CD7-CD56-MPO- | BM | 71% | |
| 092/17 | AML M2/M3 | Female/87 | N/A | CD34-CD117+/-CD13-/+ CD33+CD15-  HLA-DR- CD38+CD7-CD56- | leukapheresis | 83% | |

**Figure S1**

**
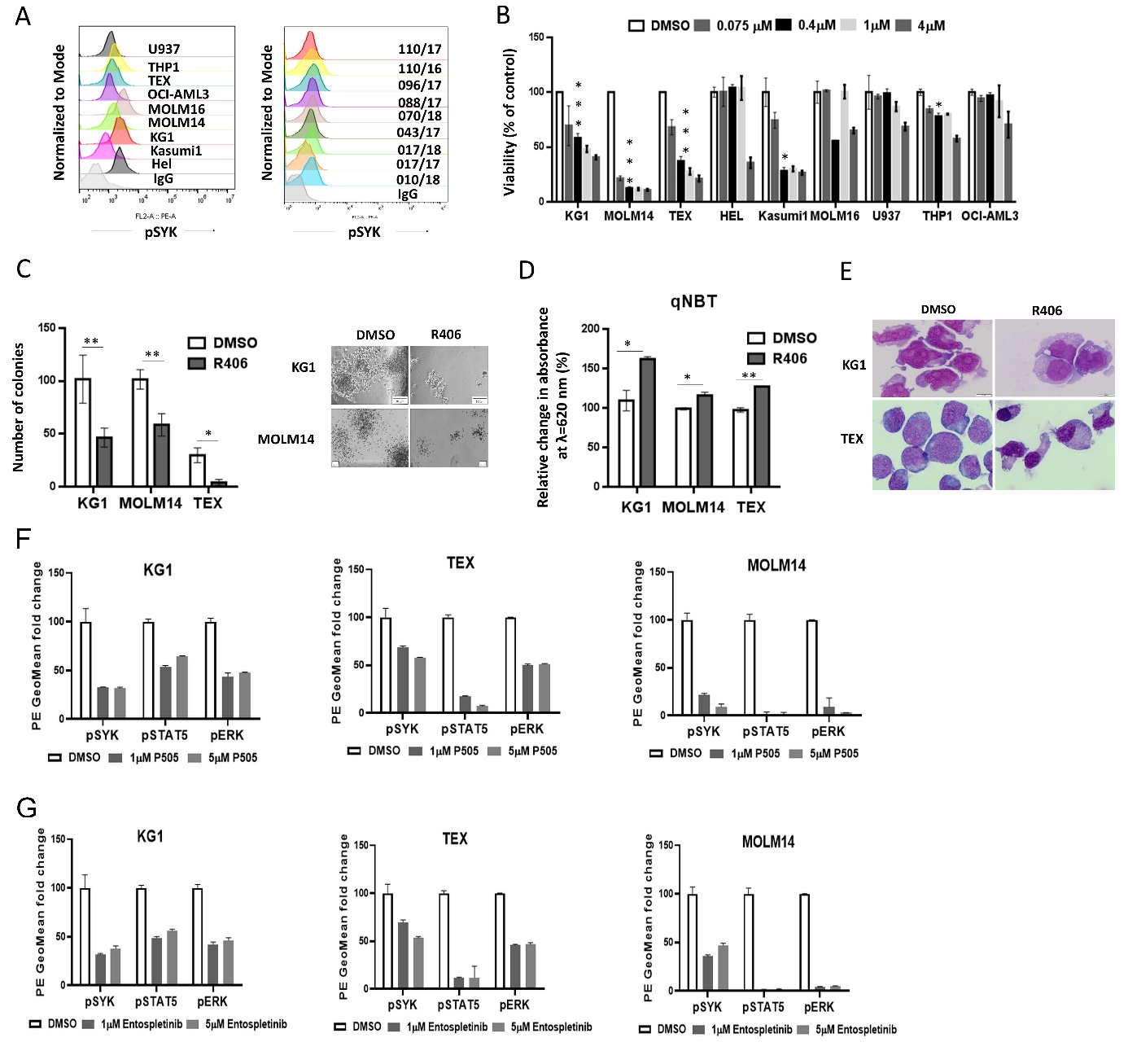
**

**Figure S1. SYK inhibitor, R406, induces differentiation and reduces viability and clonogenic potential of AML cells.**

**A.** Phospho-SYK (Tyr 352) expression in a panel of AML cell lines and primary AML blasts (n=9), assessed by phos-flow.

**B.** AML cells lines were incubated with indicated concentrations of R406 for 72h, and cell viability was assessed using MTS assay. Change in viability is presented relative to DMSO. Bars represent mean +/-SD from 4 biological replicates. Bars for KG1, MOLM14 and TEX show mean +/-SD from 2 independent experiments. Statistical analysis was performed using Student’s t-test. ** p<0.001.

**C.** KG1, MOLM14 (5×10^3^/plate) and TEX cells (35×10^3^/plate) were plated in GFH4434 medium containing either DMSO or R406. Colonies were counted under the light microscope 14 days after plating. The graph shows mean number of colonies (+/-SD) obtained from 3 independent plates.

**D.** AML cell lines were incubated for 3 days with vehicle or R406 and the cell differentiation was assessed in the NBT reduction assay. Graph indicates relative (to DMSO) change in absorbance at 620nm. Error bars indicate SD from biological replicates (n=3).

**E.** May-Grünwald-Giemsa staining of KG1 and TEX cells after 5 days of treatment with R406. Images were acquired with an Olympus CX41 microscope, under 100x objective lens (oil immersion). * p<0.05, ** p<0.01.

**F and G.** AML cells lines were incubated with indicated concentrations of P505 and entospletinib for 24h. Thereafter, activity of pSYK, pSTAT5 and pERK was assessed by phos-flow using PE-conjugated antibodies. GeoMean values after subtracting IgG GeoMean are demonstrated relative to DMSO treated cells. Bars represent mean +/-SD from 2 biological replicates.

**Figure S2**

**
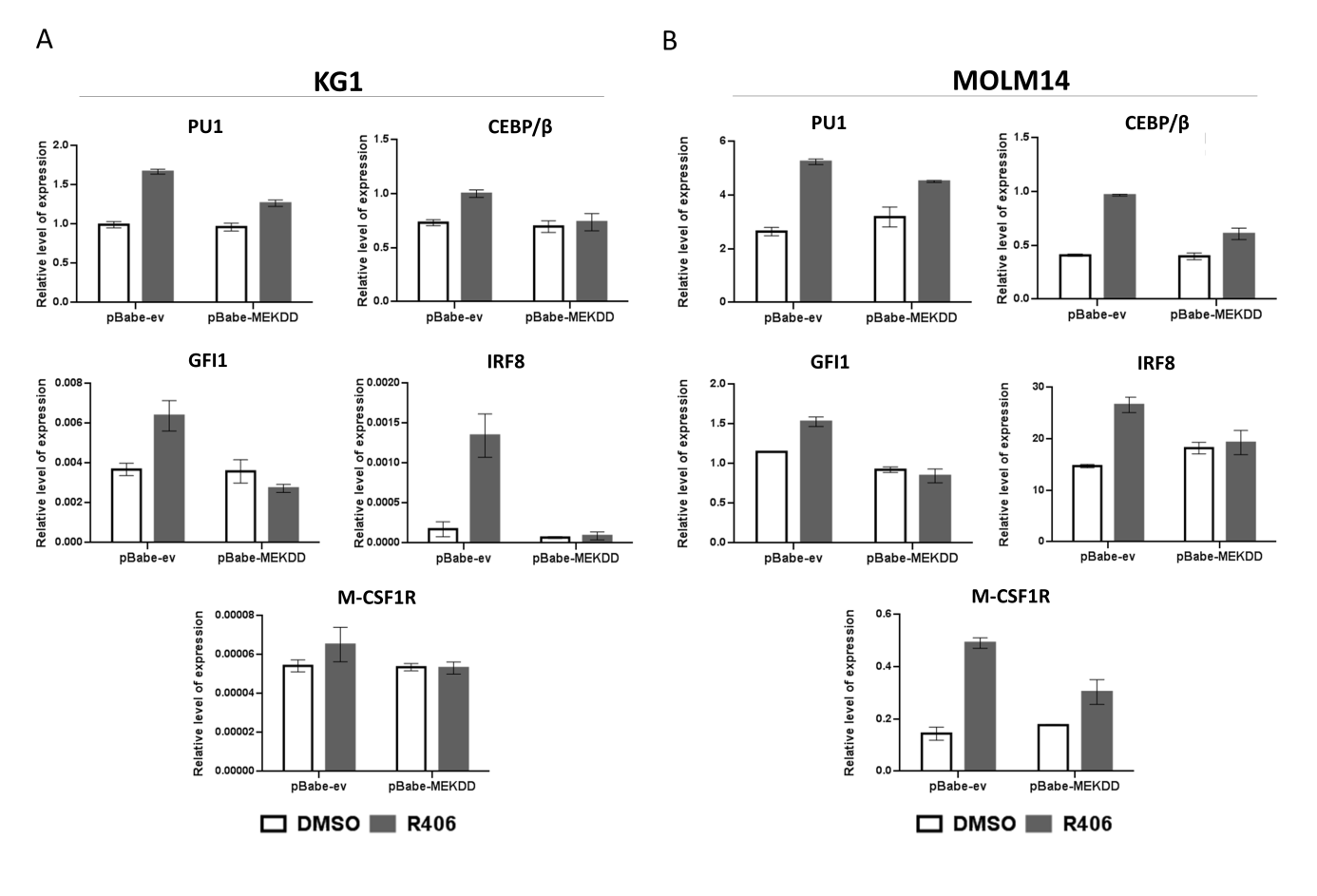
**

**Figure S2. SYK signals through MAPK/ERK1/2 pathway to block differentiation of leukemic cells.**

Expression of transcription factors involved in myeloid maturation was assessed by real-time PCR experiment (qRT-PCR) in KG1 **(A)** and MOLM14 cells **(B)** transduced either with MEKDD or empty vector after 24h of incubation with R406 using. Expression was calculated relative to a housekeeping gene (GAPDH) using 2^-ΔΔCt^ method. Error bars represents SD from technical replicates (n=3).

**Figure S3**

**
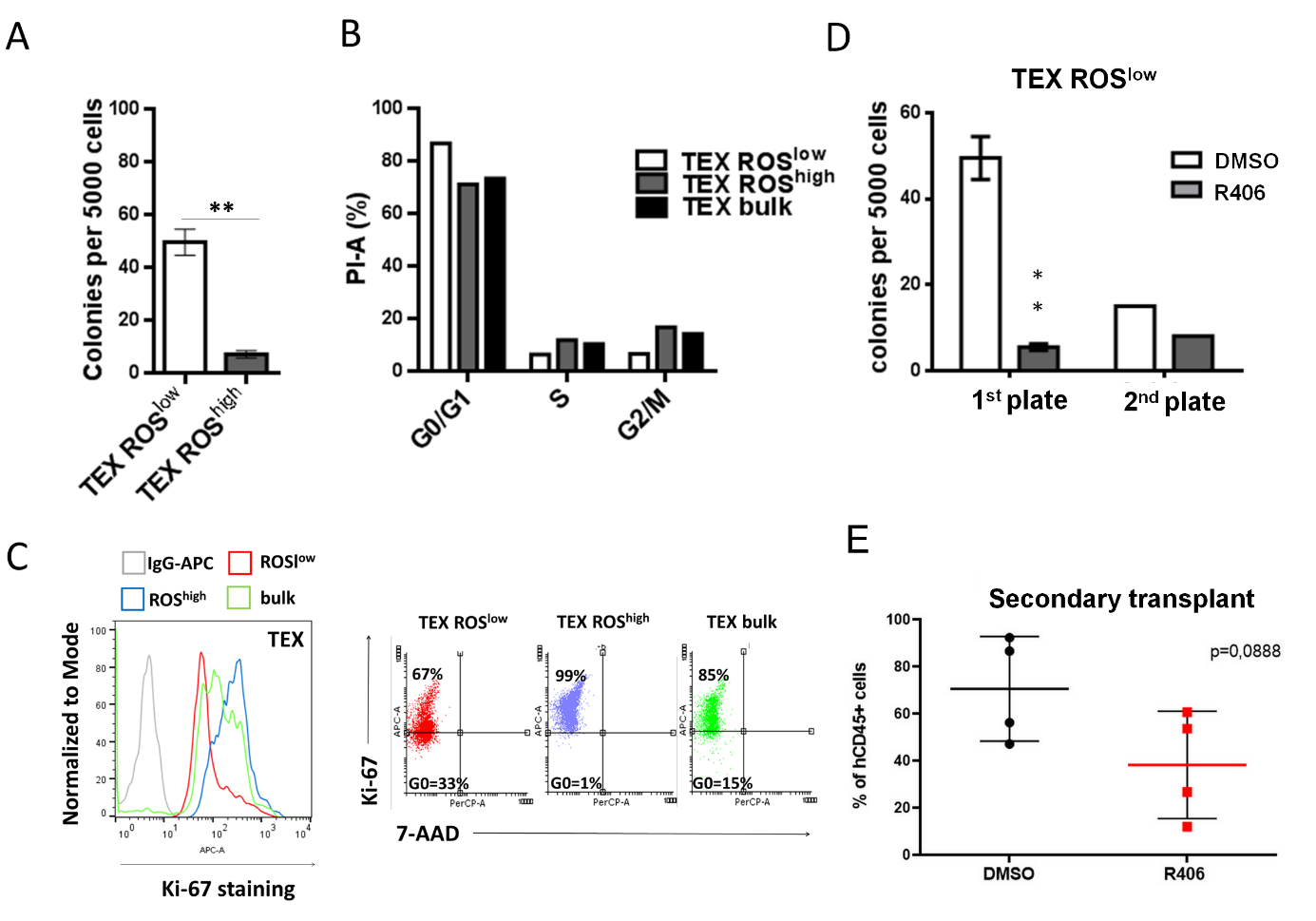
**

**Figure S3. LSC-like characteristics of TEX ROS-low cells compared to TEX ROS-high population.** TEX cells were labeled with CM-H_2_DCFDA (DCF) and then sorted to populations containing either low level of ROS (bottom 15% of dye distribution) or high level of ROS (top 15% of dye distribution).

**A.** Number of colonies per 5×10^3^ plated cells in ROS-low and ROS-high populations. Mean +/- SD values from 3 independent plates are plotted. ** p<0.01.

**B.** Cell cycle distribution of sorted ROS-low, ROS-high and bulk TEX cells performed by propidium iodide staining followed by flow cytometry.

**C.** Ki-67 fluorescence intensity in ROS-low, ROS-high subsets and bulk TEX population. Sorted cells were fixed, permeabilized and stained with Alexa Fluor 647-conjugated anti-Ki-67 antibody, and analyzed by FACS. Right panel: cell proliferation analysis in sorted ROS-low, ROS-high and bulk TEX cells. Percentage of quiescent (G0) cells in ROS-low, ROS-high fractions and bulk TEX cells is indicated.

**D.** Serial re-plating assays of the TEX ROS-low cells. Bars show mean number of colonies +/-SD from 2 independent plates (1^st^ plate) or the number of colonies (2^nd^ plate). ** p<0.01

**E. Leukemia burden in secondary transplant recipient mice.** Animals (n=4 per group) were inoculated with 1.75x10^6^ of BM cells obtained from mice that had received primary transplants of AML cells pretreated ex-vivo with R406 or DMSO. After eight weeks, peripheral blood was analyzed for the presence of hCD45+ cells using FACS. Statistical analysis was performed using Student’s t-test.

**Figure S4**

**
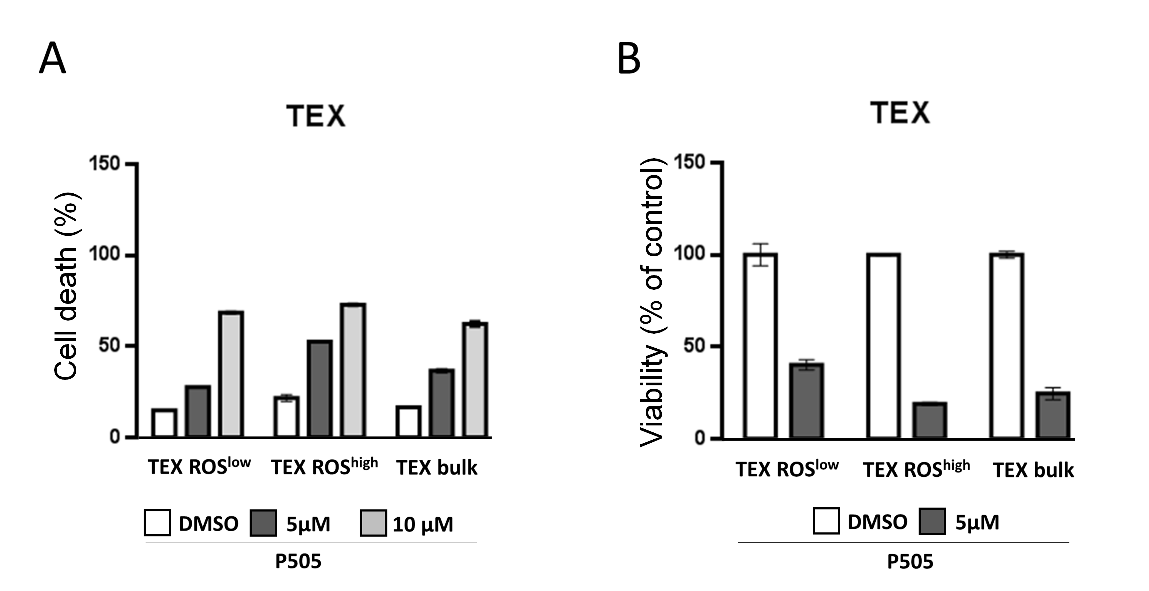
**

**Figure S4. SYK inhibition targets LSCs in AML.** Sorted ROS-low, ROS-high and bulk TEX cells subsets were treated for 3 days with vehicle or indicated doses of P505 (SYK inhibitor). Thereafter, cells were stained with PI to assess cell death by flow cytometry **(A)**, or analyzed by MTS assay to assess cell viability **(B)**. In panel A, the percentage of PI-positive cells is shown. The graphs show mean +/-SD from 3 biological replicates.

**Figure S5**

**
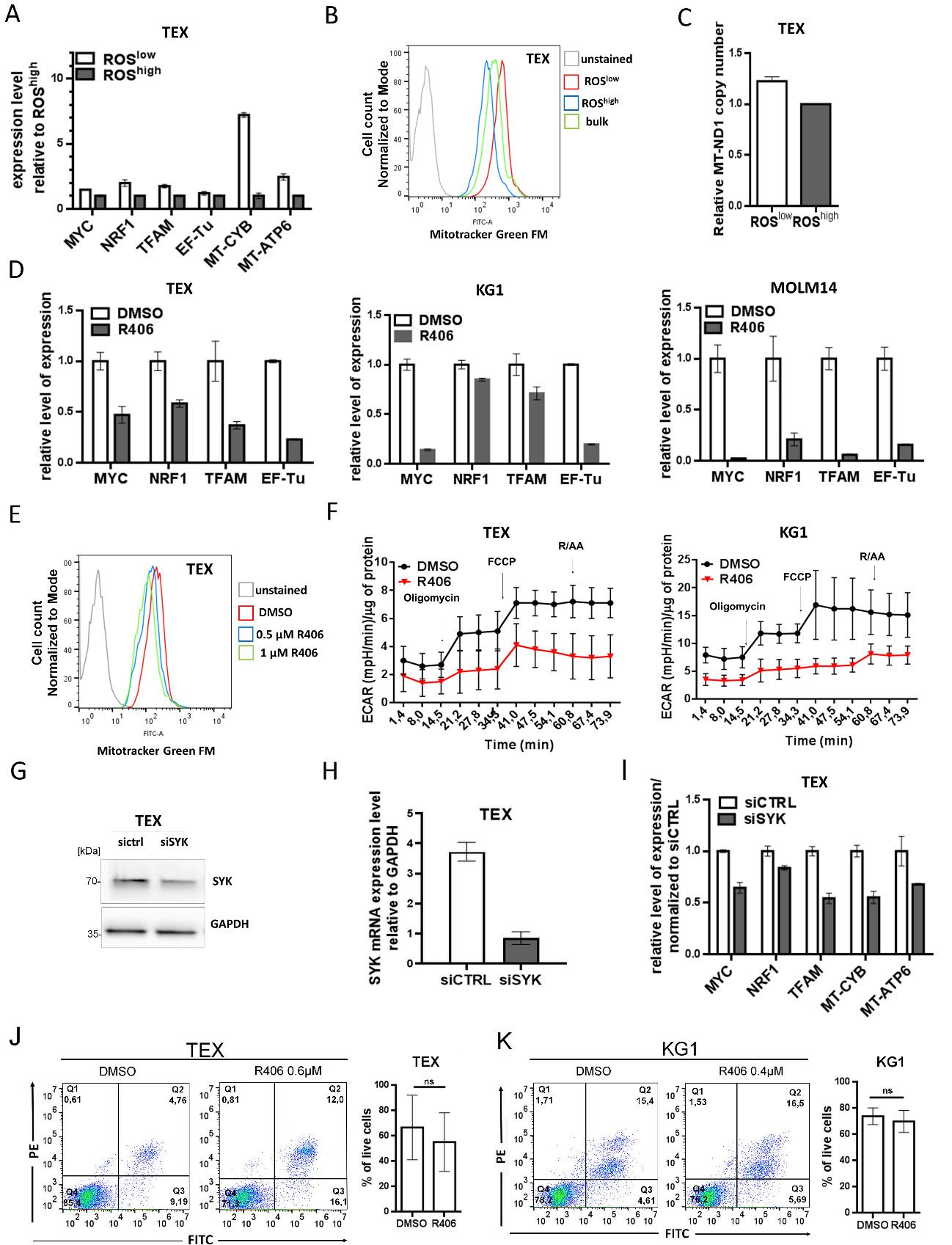
**

**Figure S5. SYK inhibition decreases mitochondrial biogenesis and OXPHOS in AML cells** (data supplemental to Figure 5).

**A.** Basal mRNA expression level of MYC, TFAM, NRF1, EF-Tu, MT-CYB and MT-ATP6 in TEX ROS-low and TEX ROS-high subsets. Expression was determined by qPCR using 18s RNA as an internal standard. Mean +/- SD from 4 replicates well is shown.

**B.** Cells were stained with DCF and sorted into two subpopulations – characterized by either low or high fluorescence. After 3 days in culture, mitochondrial mass in sorted ROS-low, ROS-high and bulk TEX subsets was determined by Mitotracker Green FM staining, followed by flow cytometry analysis. Unstained control was prepared by mixing TEX ROS-high, ROS-low, and bulk cells in a 1:1:1 ratio.

**C.** Mitochondrial DNA copy number in sorted ROS-low and ROS-high TEX subsets. DNA was extracted from sorted TEX ROS-low and TEX ROS high cells and abundance of mitochondrial gene ND1 was determined by qPCR and normalized to human single-copy globulin (HGB) gene abundance. ND1/HGB ratio in TEX ROS-high cells was arbitrarily set to 1; Bars show mean +/- SD of 3 technical replicates.

**D.** mRNA expression level of MYC, NRF1,TFAM, EF-Tu in TEX, KG1 and MOLM14 cells treated for 24h with DMSO or R406 (1 µM). Expression was determined by qPCR using 18s RNA as an internal standard. Graph shows mean +/- SD of 3 technical replicates.

**E**. Mitochondrial mass in TEX cells cultured for 24h with DMSO or indicated concentrations of R406 as determined by Mitotracker Green FM staining and flow cytometry analysis.

**F.** Extracellular acidification rate (ECAR) in TEX and KG1 cell after 24h incubation with R406 (0.6 µM, 0.4 µM, respectively). Arrows denote addition of oligomycin (1 µg/mL), FCCP ( TEX 1.5 µM and KG1 1.25 µM), and rotenone and antimycin A mix (R/AA, 1 µM each). Values shown are average of 3 (KG1) or 4 (TEX) independent experiments +/- SEM.

**G and H.** SYK silencing with siRNA. 10^6^ TEX cells were transduced with 1µM of SYK-targeting siRNA, and after 72h of incubation protein extracts were obtained and immunoblotted with anti-SYK antibody. SYK mRNA expression level was determined using RT-qPCR.

**I.** Effects of siSYK on mRNA expression level of MYC, NRF1, TFAM, EF-Tu, MT-CYB and MT-ATP6 in TEX cells incubated for 96 hrs with siRNA. Transcript levels were determined by qPCR and normalized to 18S. Normalized mean values of siSYK treated cells as compared to non-targeting oligos-treated controls (siCTRL) (presented as equal to 1) are shown. Error bars indicate SD from technical replicate wells (n=3).

**J and K.** TEX and KG cells were incubated 24h with vehicle or R406 (0.6μM or 0.4μM, respectively). Cell death was assessed by AnnexinV-FITC/PI staining. Representative scatterplots are shown. Bar charts show mean values +/-SD of 2 independent experiments. Statistical analysis was performed using Student’s t-test, ns: not significant.

**Figure S6**


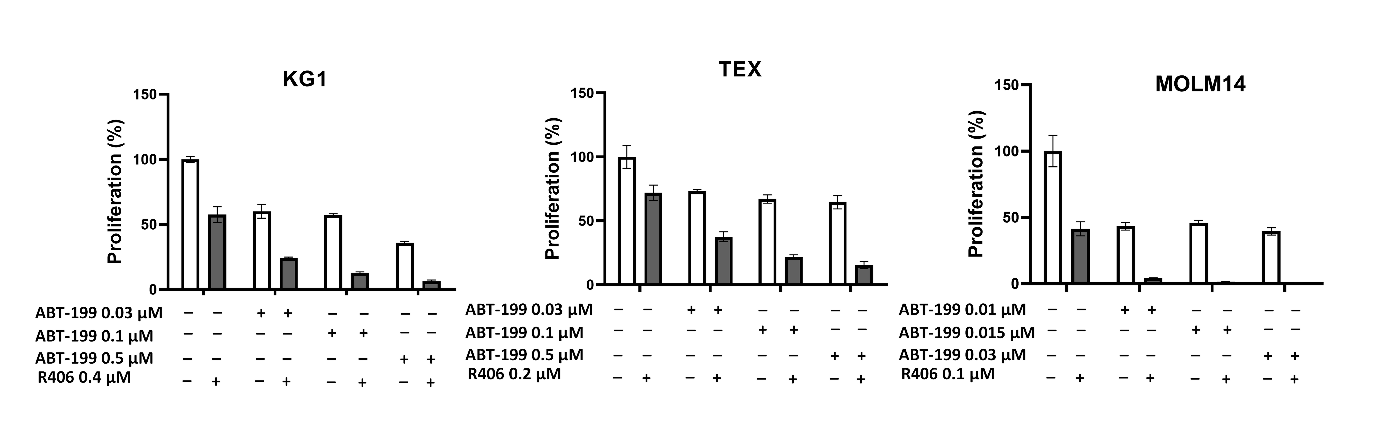


**Figure S6. R406 sensitizes AML cells to ABT-199 BCL2 inhibitor.** KG1, TEX and MOLM14 cells were treated for 3 days with vehicle, indicated doses of ABT-199, R406 or combination of both. Thereafter, cell viability was assessed by MTS assay. Bars represent SD from 3 biological replicates.
