# Supplementary figures and images for: SYK inhibition targets acute myeloid leukemia stem cells by blocking their oxidative metabolism
Source: Cell Death Dis. 2020 Nov 6;11(11):956. doi: 10.1038/s41419-020-03156-8 (PMC7648638; doi:10.1038/s41419-020-03156-8)

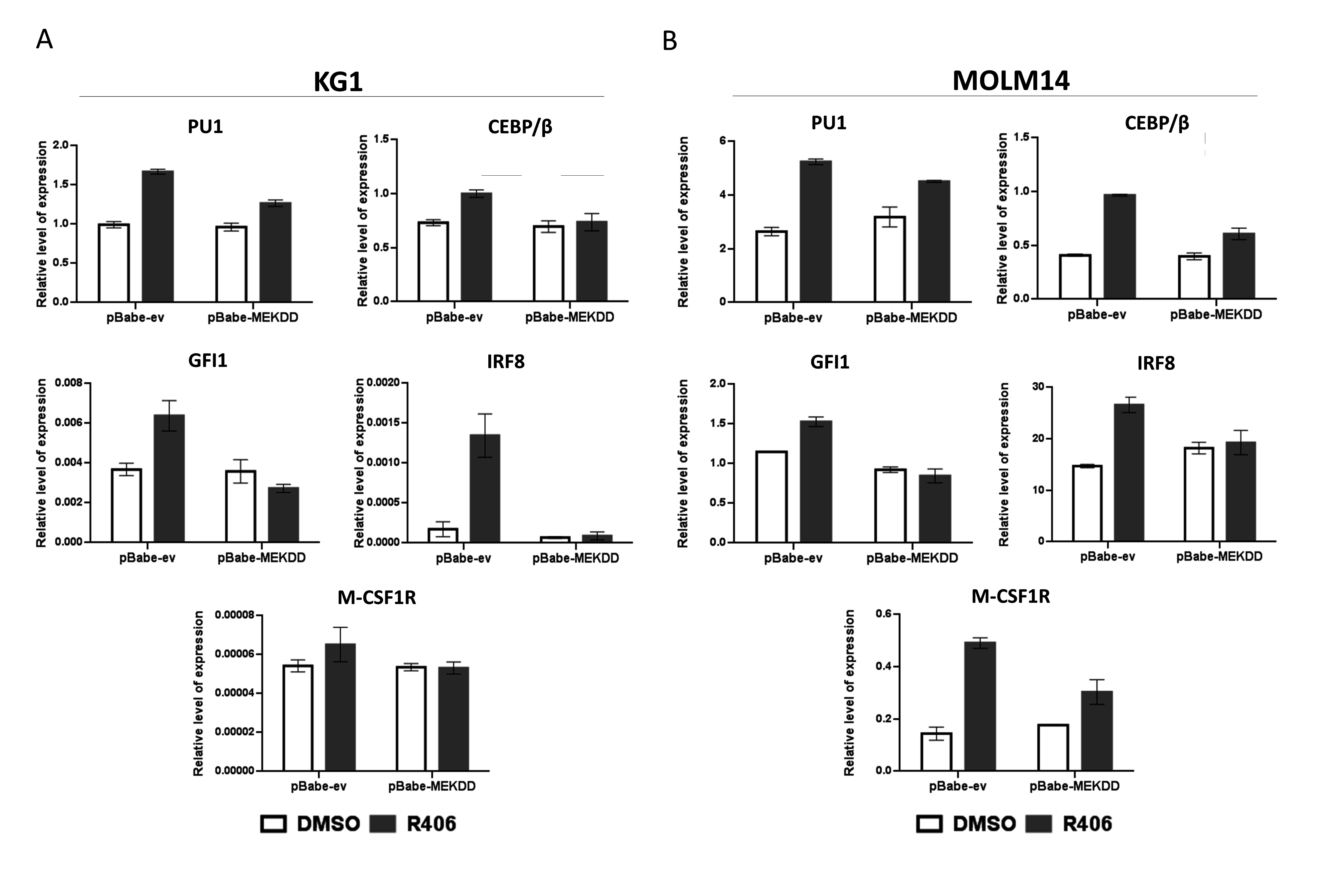

Supplement: Supplementary file 3 — Supplementary Figure 2 [file 41419_2020_3156_MOESM3_ESM.tif]

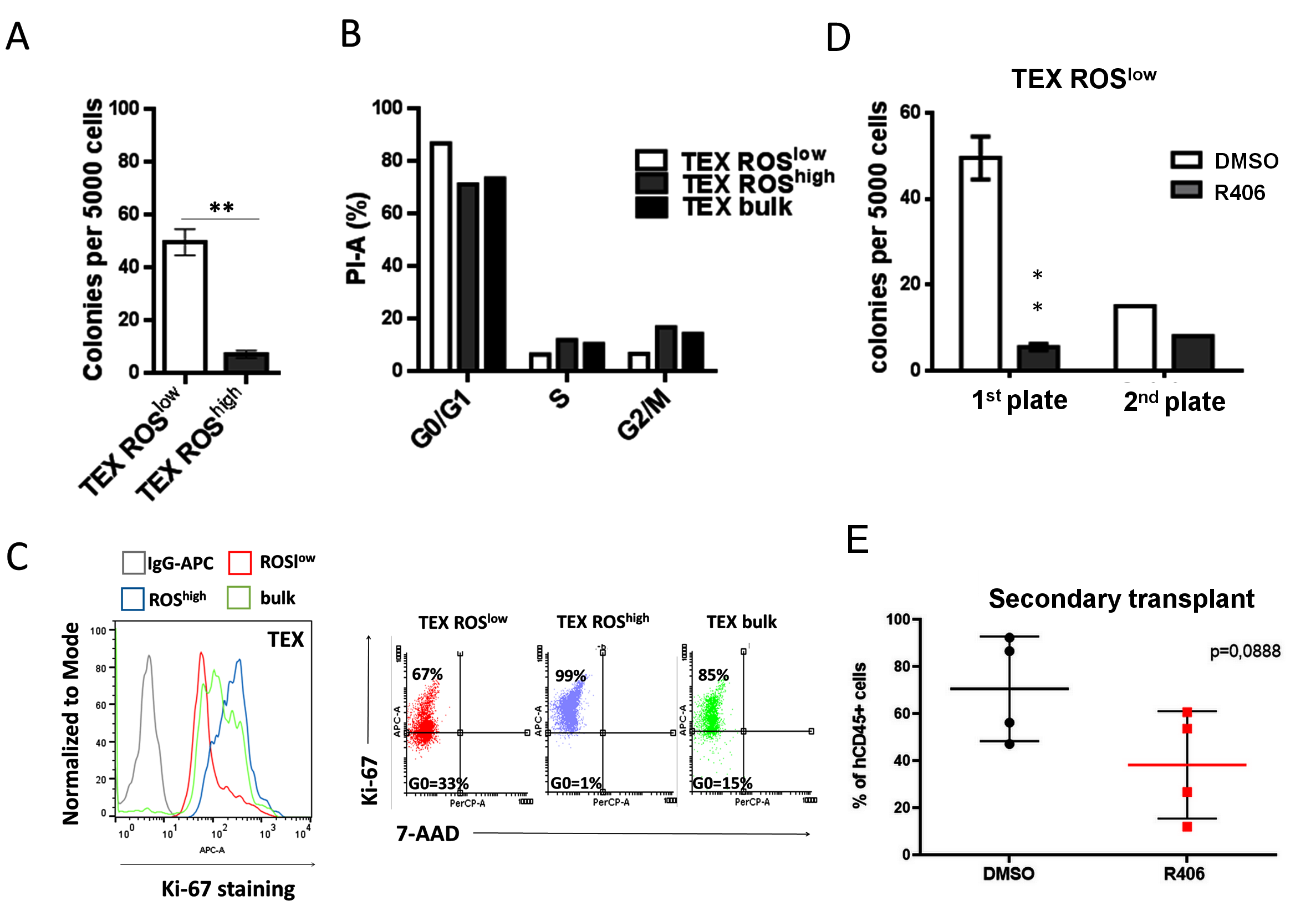

Supplement: Supplementary file 4 — Supplementary Figure 3 [file 41419_2020_3156_MOESM4_ESM.tif]

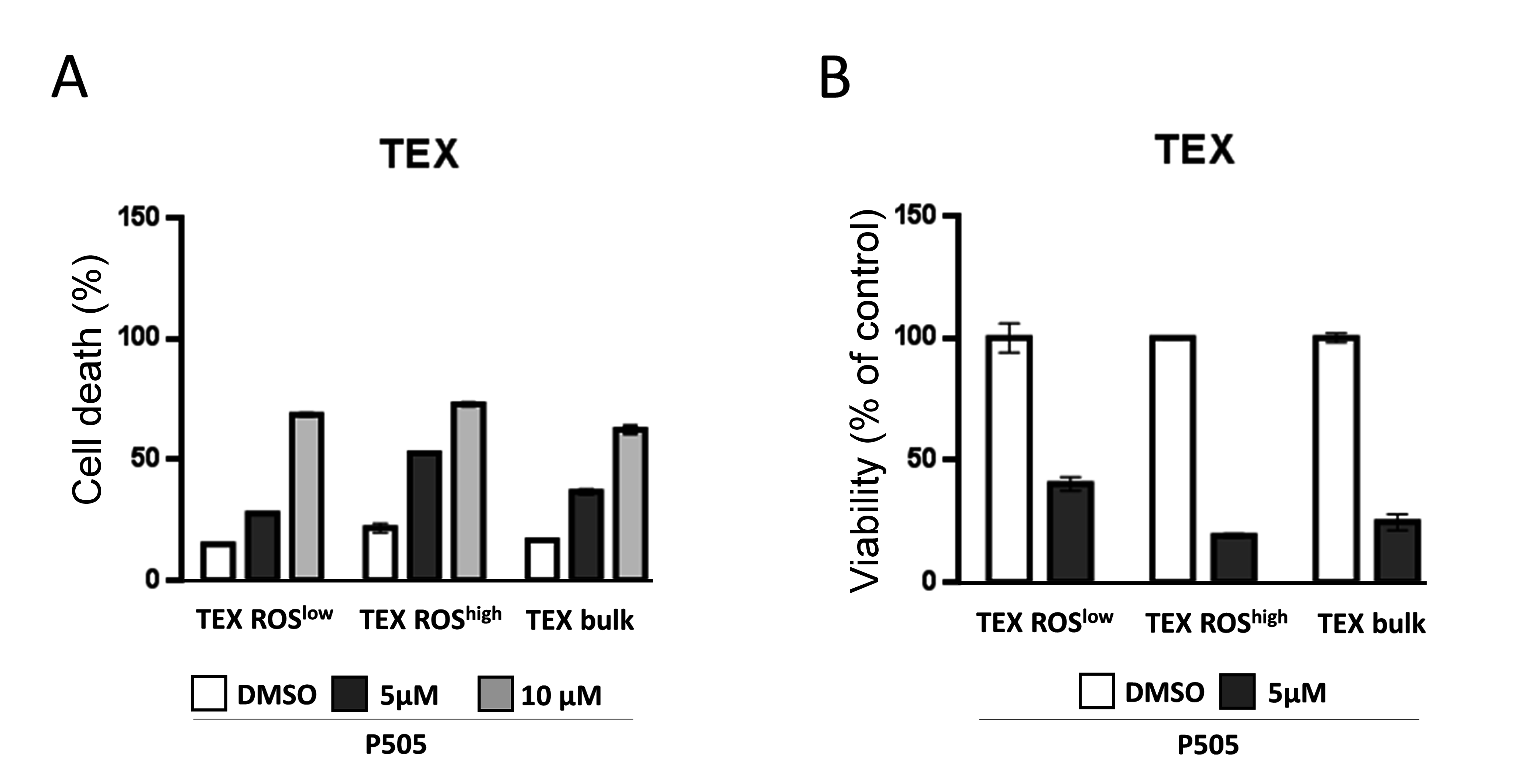

Supplement: Supplementary file 5 — Supplementary Figure 4 [file 41419_2020_3156_MOESM5_ESM.tif]

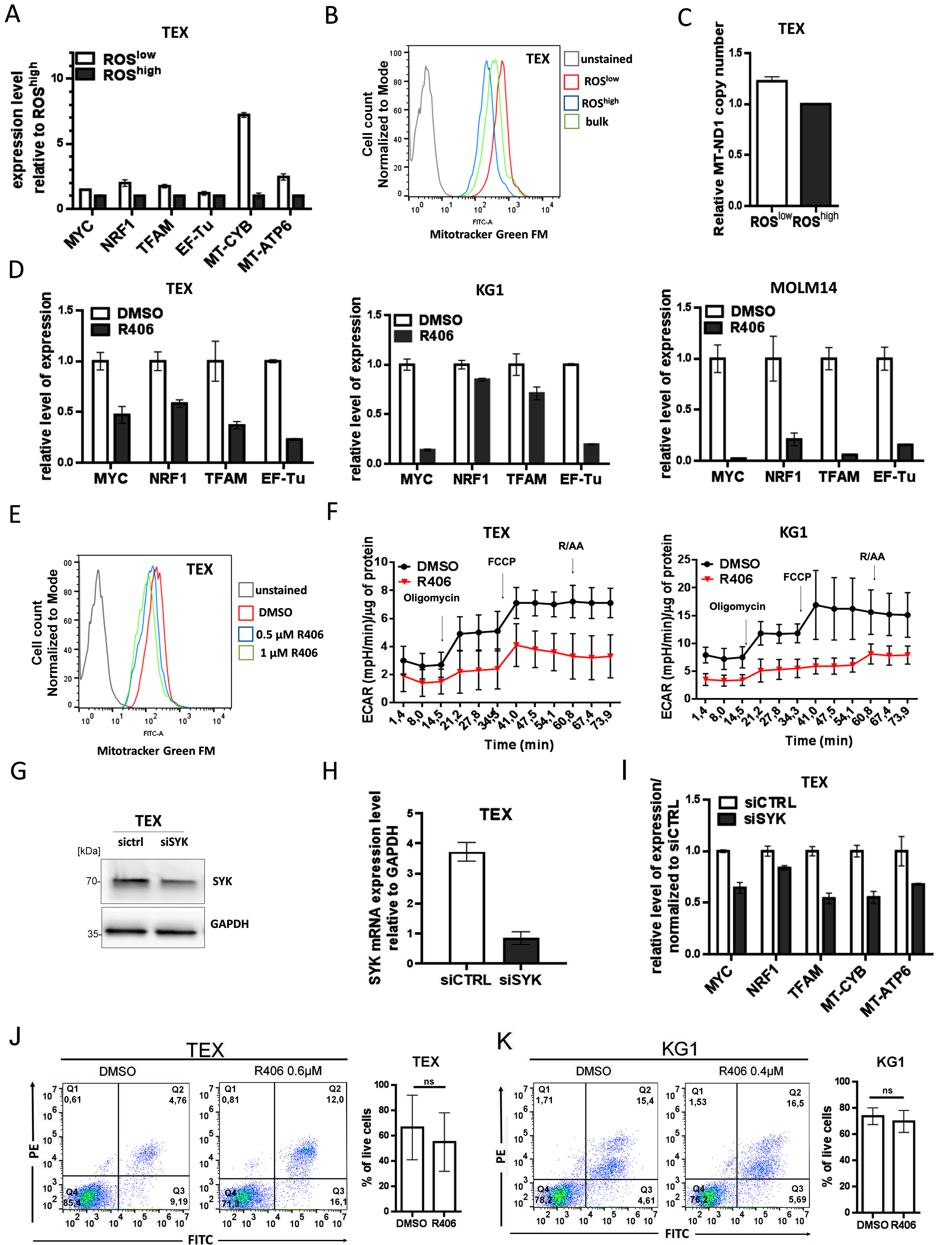

Supplement: Supplementary file 6 — Supplementary Figure 5 [file 41419_2020_3156_MOESM6_ESM.tif]

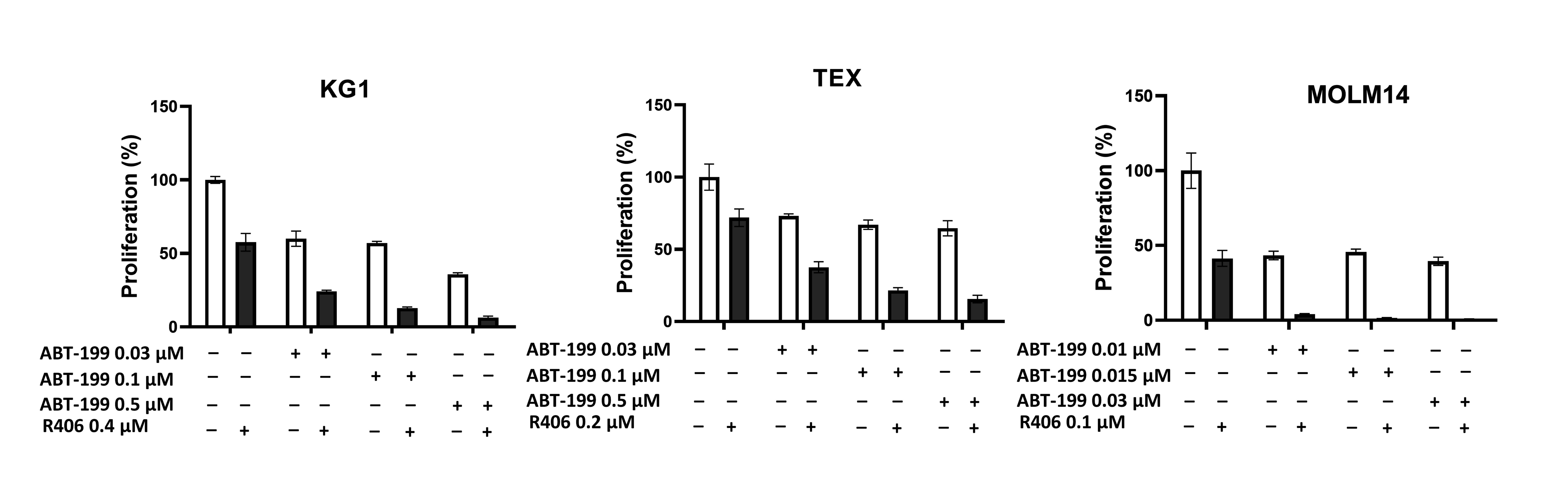

Supplement: Supplementary file 7 — Supplementary Figure 6 [file 41419_2020_3156_MOESM7_ESM.tif]
